# The characterization of a rhamnosylated triterpenic saponin from *Quillaja saponaria* Mol. reveals novel adjuvant, antibacterial, antiviral, antifungal and antiproliferative properties

**DOI:** 10.3389/fphar.2026.1856067

**Published:** 2026-08-26

**Authors:** Roberto Bobadilla-Fazzini, María José Marchant, Elisabeth Grohmann, Leda Guzmán, Leandro Padilla

**Affiliations:** 1 Desert King Chile, Valparaíso, Chile; 2 Laboratorio de Química Biológica, Instituto de Química, Facultad de Ciencias, Pontificia Universidad Católica de Valparaíso, Valparaíso, Chile; 3 Department of Microbiology, Faculty of Life Sciences and Technology, Berlin University of Applied Sciences, Berlin, Germany

**Keywords:** adjuvant, antibacterial, antifungal, antiproliferative, antiviral, Quillaja, saponin, vaccine

## Abstract

Quillaja: is an abundant source of triterpenic saponins, glycosides with immunostimulant properties, currently employed as vaccine adjuvants. Although current research and commercial applications have focused on the most abundant saponins in *Quillaja* extracts (QS-7, QS-17, QS-18 and QS-21), the vast chemical variety in *Quillaja* saponins remains largely unexplored. The discovery of new saponins suited as vaccine adjuvants would open the door to a wider supply of adjuvants in the future. This paper describes the isolation and chemical characterization of Saponin FR, a new saponin fraction purified from extracts of a *Quillaja* specimen depleted of traditional saponins QS-7, QS-17, QS-18 and QS-21. The main component of this fraction is a bidesmosidic glycoside of quillaic acid substituted at C-3 and C-28 (molecular mass 2003.93 Da). Mass and NMR spectroscopies characterization revealed a structure similar to QS-21 where the 3-O-[β-d-xylopyranosyl-(1→3)-[β-d-galactopyranosyl-(1→2)]-β-d-glucuronopyranoside] is replaced by a 3-O-[α-l-rhamnopyranosyl-(1→3)-[β-d-galactopyranosyl-(1→2)]-β-d-glucuronopyranoside] substituent at position C3. Tests in mice immunized with ovalbumin demonstrated that Saponin FR induces a tenfold increase of the total immunoglobulin titer compared to the antigen control without Saponin FR (mostly IgG1 type). Cytokine analysis of serum samples showed a significant increase of IFN-γ upon immunization with Saponin FR. By contrast the production of cytokines IL-4, IL-6 and IL-10 was not observed, suggesting a pro-inflammatory Th1 response. Regarding the antimicrobial effect of Saponin FR, *S. aureus* ATCC25923 and *K. pneumoniae* ATCC 1705 were susceptible to concentrations as low as 0.12 µM and the same effect was observed against a clinical isolate of *C. albicans*. Saponin FR was also effective as antiviral agent evaluated in *in vitro* assays using HeLa cells infected with the human adenovirus 5 (VR-5), significantly reducing its infectivity at concentrations as low as 1 nM in *in vitro* assays. Finally, Saponin FR showed remarkable antiproliferative activity against gastric (AGS 1739) and cervix (HeLa CRM-CCL-2) adenocarcinoma cell lines with half maximal inhibitory concentrations (IC_50_) of 2.9 and 2.6 µM, respectively. Overall, Saponin FR broadens the spectrum of *Quillaja* saponins for new vaccine adjuvant, antimicrobial, antiviral and antitumoral formulations with potential clinical application.

## Introduction

Saponins are steroid or triterpenoid glycosides abundant in the plant kingdom having a wide range of medicinal and commercial uses (Hostettmann and Marston, 1995). The Chilean tree *Quillaja saponaria* is an abundant source of triterpenic saponins (Montenegro et al., 2001). Although *Quillaja* saponins have been used as a surfactant in cosmetic and food industry (Fleck et al., 2019), there is an increasing interest in using them as components of antibacterial, antiviral, antifungal, as well as anti-tumoral formulations, and particularly for immunological adjuvants, substances able to induce a strong and prolonged humoral (Th2) and cellular immunity (Th1) (Magedans et al., 2019). *Quillaja* saponin mixtures have exhibited a low minimum inhibitory concentration (100 μg/mL) against different pathogenic microorganisms, such as *Staphylococcus aureus*, *Salmonella typhimurium*, and *Escherichia coli* (Hassan et al., 2010; Kaper et al., 2004). Roner et al. (2007) demonstrated antiviral activity of *Quillaja* saponins extracts against vaccinia virus, herpes simplex virus type 1, varicella zoster virus, human immunodeficiency viruses 1 and 2 (HIV-1, HIV-2) and reovirus, indicating that the extract can alter viral envelopes and capsid proteins and prevents virus attachment to the host cell. *In vitro* studies showed that saponin-rich *Quillaja* extracts inhibit the growth of common phytopathogenic fungi including *Pythium ultimum*, *Fusarium oxysporum*, and *Verticillium dahlia* (Chapagain et al., 2007). These antimicrobial and antiviral activities are related to the tensoactive properties of *Quillaja* saponins (Berlowska et al., 2015). Additionally, the *Quillaja* saponin QS-21 and its aglycone quillaic acid also have cytotoxic activity against gastric cancer cell lines (Guzmán et al., 2020).

Subunit vaccine technology based on high purity antigens, avoiding the use of attenuated and inactivated pathogens, has as a major drawback in its weak immunogenicity and the lack of an effective immune response induction when used alone (Marciani, 2018; Wang and Xu, 2020). This holds particularly true for vulnerable groups such as infants and the elderly (Simon et al., 2015). The outstanding capacity of *Quillaja* saponins in stimulating both humoral and cellular immunity (Dalsgaard, 1978; Chavali et al., 1987) opened the way to the development of saponin-based adjuvants for those vaccines (Marciani, 2018). In this sense, *Quillaja* saponins are known to be able to activate both Th1 and Th2 responses, providing a balanced and enhanced immune response. As previously described, Th2 humoral immune response mediated by the cytokine interleukin 4 (IL-4) preferentially induces switching to IgG1, whereas Th1 cell-mediated immune response driven by cytokines such as interferon gamma (INF-γ) result in IgG2A, IgG2B, and IgG3 switching (Snapper and Paul, 1987).

So far, academic and commercial attention has been paid towards the most abundant saponins in the mixture of more than 100 saponins in *Q. saponaria* (Kite et al., 2004), with the saponins QS-7, QS-17, QS-18 and QS-21 (Kensil et al., 1991) as the most conspicuous.

Currently, QS-21 is being employed as an ingredient of the adjuvant system 01 (AS01) in the recombinant subunit commercial vaccines for preventing herpes zoster (Singh et al., 2020), malaria (Syed, 2022) and recently respiratory syncytial virus (Schwartz et al., 2024). Less pure fractions enriched in QS-7 and QS-21 are employed in the adjuvant Matrix-M^TM^ present in the vaccine NVX-CoV2373 against the SARS-CoV-2 virus causing COVID-19 disease (Underwood et al., 2023) and the influenza vaccine qINV - in phase 3 stage (Shinde et al., 2022).

As mentioned earlier there is a huge chemical variability in the triterpenic saponins from *Q. saponaria*, associated mostly to the different glycosylation/acylation pattern of the quillaic acid aglycone particularly at the C-28 position (Kite et al., 2004). Currently, the vast chemical variability in *Quillaja* saponins remains largely unexplored for pharmaceutical use. In the context of emerging diseases and pandemics, there is an urgent need for the discovery of new saponins in *Q. saponaria* suitable as vaccine adjuvants to expand the yield of these components and improve their use as a resource in the pharmaceutical supply.

By an extensive screening of *Quillaja* trees, our research group has identified specimens depleted of QS-7, QS-17, QS-18 and QS-21, and enriched in rhamnosylated saponins normally found in low concentrations in *Quillaja* extracts (Padilla et al., 2022).

To explore the potential of these uncommon saponins, we isolated new saponin fractions by chromatography and studied their biological activities (immunostimulant, cytotoxic as well as antimicrobial). Characterization of the biological activities of those new fractions is critical since minor structural variations in the oligosaccharide chains in C-3 and -28 positions would generate non-predictable and significant changes in the biological activity of *Quillaja* saponins, particularly in their toxicity and adjuvant activity (Sun et al., 2009). The best example is QS-18, the most abundant saponin in *Quillaja* extracts (Kensil et al., 1991), which differs from QS-21 just in an additional glucose at the oligosaccharide chain linked to the C-28 position (Kite et al., 2004). Although both QS-18, and QS-21 had adjuvant activity (Kensil and Marciani, 1991), QS-18 was lethal to 4 of 5 mice at doses as low as 125 μg, while QS-21 shows only some lethality at 500 µg (Kensil et al., 1991).

This paper describes the purification, characterization and biological activity of Saponin FR (fraction rhamnosylated), one of the rhamnosylated saponins found in a specific *Q. saponaria* Molina plant that can be reproduced vegetatively, and that is depleted of QS-7, QS-17, QS-18 and QS-21.

## Materials and methods

### Biological and chemical reagents

*Quillaja saponaria* Molina bark and/or lignified twigs were obtained from plants grown by Desert King Chile S.A. All chemical reagents and solvents employed in the study were analytical grade unless specifically stated otherwise.

Cell lines and virus: The human gastric cancer cell line AGS (ATCC 1739) and human cervical adenocarcinoma cell line–HeLa (ATCC CRM-CCL-2) were obtained from the American Type Culture Collection (ATCC) (Manassas, VA, United States) and were maintained in Dulbecco’s Modified Eagle’s Medium (DMEM; Gibco, United States) supplemented with 10% fetal bovine serum (FBS; Gibco, United States), 100 U/mL penicillin, and 100 μg/mL streptomycin. Cells were cultured in a humidified 5% CO_2_ incubator at 37 °C. The human gastric epithelial cell line (GES-1) was used as healthy control, maintained in DMEM medium (kindly donated by Dr. Dawit Kidane-Mulat from the University at Texas-Austin, United States). The Human Adenovirus Type 5 (VR-5; ATCC VR-5) was obtained from ATCC. The stocks were harvested, clarified by low-speed centrifugation (3000 × *g*, 15 min), aliquoted, and stored at −80 °C. Bacterial strains *Staphylococcus aureus* ATCC 25923 and *Klebsiella pneumoniae* ATCC 1705 were purchased from Microbiologics® (Microbiologics, MN, United States). Methicillin-resistant *S. aureus* (MRSA) PUCV Doc 1505 strain and *Candida albicans* ATCC 14053 from the American Type Culture Collection, United States, were donated by Dr. Levicán from the Microbiology Laboratory from Medical Technology School, Pontificia Universidad Católica de Valparaiso. Trypticase soy agar (TSA), tryptic soy broth (TSB), Mueller-Hinton broth (MHB), Mueller–Hinton agar (MHA), and potato dextrose agar (PDA) were purchased from Oxoid (ThermoFisher Scientific, Waltham, MA, United States). Kanamycin, methicillin and fluconazole were purchased from Sigma-Aldrich (St. Louis, MO, United States). The McFarland turbidity standard was acquired from BioMérieux. MTT (3-(4,5-dimethylthiazolyl-2)-2,5 diphenyltetrazolium bromide) was purchased from Sigma-Aldrich (St. Louis, MO, United States).

### Isolation of saponin fractions

Crude saponin extract (water solid ratio of 6:1, 60 °C for 4 h in deionized water- Milli-Q grade) was prepared from selected *Q*. *saponaria* lignified twigs previously dried at 60 °C for at least 12 h and milled. The crude saponin extract was concentrated 8 times by evaporation at 60 °C, frozen and freeze-dried for 48 h. The obtained solid fraction was dissolved in methanol (1:10), centrifuged for 15 min at 2,650 × *g* and filtered onto 0.45 µm sterile nylon filter. The remaining solids were washed with methanol, centrifuged and filtered as before, pooling together all the filtered solutions. The pool of solutions was concentrated at 60 °C and 90 rpm in a rotary evaporator. This concentrated methanol extract was processed through two chromatographic steps. In the first step, aliquots of the liquid methanol extract were injected in a semipreparative reverse phase octadecylsilane HPLC column (inner diameter 21.2 mm; length 250 mm; particle size 7 µm) and eluted in gradient mode at constant flow rate (20 mL/min) using a gradient elution with water and methanol (both with formic acid 0.1% v/v). The eluate was recovered as discrete fractions; the saponin profile of the collected fractions was determined off-line in a Waters Acquity H-Class UPLC chromatography system coupled to a Waters PDA Acquity diode array absorbance detector. The analytical column used was an Acquity UPLC® BEH C-18 (particle size 1.7 µm, inner diameter 2.1 mm; length 100 mm) (Waters, MA, United States). The column was maintained at 30 °C during each chromatographic run. The injected samples were eluted at constant flow rate (0.41 mL/min) in gradient elution mode employing the following mobile phases: Solvent A: *Ortho*-phosphoric acid 0.15% v/v in water, and Solvent B: *Ortho*-phosphoric acid 0.15% v/v in acetonitrile. The solvent gradient employed is described as follows: Step 1: 0.0–5.0 min from 34% to 45% of Solvent B; Step 2: 5.0–7.0 min 45% of Solvent B; Step 3: 7.0–10.0 min from 45% to 34% of Solvent B, and Step 4: 10.0–15.0 min 34% of Solvent B. The eluted peaks were detected by monitoring the absorbance at 210 nm. Fractions containing Saponin FR were identified as the precedent peak of saponin QS-21, normally considered an impurity in standard *Quillaja* extracts, and selected for further recovery. The selected fractions of the preparative runs were pooled together and vacuum-concentrated in a rotary-evaporator. The residual aqueous solution obtained was frozen and freeze-dried resulting in the “intermediate FR”. For the second chromatographic separation step, the “intermediate FR” was dissolved in 30% acetonitrile in water to 100 g/L, sonicated for 20 min and centrifuged at 20,000 × *g* for 15 min. One hundred (100) μL of this solution were injected in a semipreparative reverse phase octadecylsilane HPLC column (inner diameter 21.2 mm; length 250 mm; particle size 7 µm) and eluted in gradient mode at constant flow rate (17 mL/min) using a gradient elution with water and acetonitrile (both with formic acid 0.1% v/v). The eluate was recovered as discrete fractions and their saponin profiles were determined off-line by UHPLC as described before. Fractions containing Saponin FR were selected for further recovery of the product. The selected fractions of preparative runs were pooled together and vacuum-concentrated in a rotary-evaporator. The residual aqueous solution was frozen and freeze-dried, rendering approximately 100 mg of Saponin FR per Kg of selected *Q. saponaria* lignified twigs.

### Liquid chromatography, NMR and mass spectrometry analysis

Saponin samples were characterized by proton and carbon-13 nuclear magnetic resonance in a Bruker Avance III HD-400 spectrometer. Chemical shifts are expressed in parts per million (δ scale) referenced to the protium residue of the DMSO-*d*_6_ (CD_3_SOCD_3_) employed as NMR solvent (δ 2.50 for ^1^H NMR, δ 40.4 for ^13^C NMR). Both samples (15 mg each) were dissolved in 0.5 mL of deuterated DMSO-*d*_6_ and analyzed by ^1^H and ^13^C NMR as well as by 2D NMR (COSY and HSQC). All the analyses were performed at 25 °C. LC-MS/MS analyses were performed in a UHPLC Elute LC series (Bruker Daltonics, MA, United States) equipped with an analytical column Kinetex™ C-18 (Phenomenex, CA, United States), using a gradient elution with water and acetonitrile (both with formic acid 0.1% v/v), coupled to a Compact QqTOF (Bruker Daltonics, MA, United States) with a high-resolution electrospray ionization source. Extracted compound chromatograms were processed employing Bruker Compass Data Analysis 4.4 Software.

### Immunogenicity assays *in vivo*

Mouse immunogenicity studies were conducted fulfilling the US NRC guidelines for the care and use of laboratory animals. Briefly, female BALB/c mice (8–12 weeks old, n = 7 per group) were immunized by intraperitoneal injection with four doses spaced 7 days apart (0, 7, 14 and 21 days), each containing 50 μg ovalbumin (OVA) and 25 μg of QS-21 or Saponin FR in 100 μL of phosphate buffer saline solution (PBS). A control was included with 50 μg OVA without saponin using the same volume of PBS. Three days after the last immunization the mice were sacrificed. Blood was obtained by intracardiac puncture. The serum was separated by centrifugation at 10,000 × *g* for 5 min at 4 °C. Antibody production was determined by ELISA assay, attaching OVA (100 ng/well) to 96-well plates for 18 h at 4 °C. Non-specific binding was avoided by washing with PBS 0.1% and Tween-20 solution. The samples were sequentially incubated with sera dilutions (1/200, 1/400, 1/800 and 1/1600), and detected with anti-mouse Ig antibody (total IgGs including subtypes IgG1, IgG2A and IgG2B) conjugated with horseradish peroxidase in the presence of 3,3′,5,5′-tetramethylbenzidine as chromogenic substrate. The antibody production was evaluated by measuring optical density at 450 nm in a plate reader (BioTek, VT, United States. In addition, the analysis of cytokines IFN-γ and TNF-α (Th1) and IL-4, IL-6 and IL-10 (Th2) in sera was done by flow cytometry (FACSCanto II, BD, NJ, United States) using the Mouse Inflammation and Mouse Th1/Th2 Cytokine Cytometric Bead Array Kits (BD, NJ, United States) running the kits standards and cytometer setup controls on each experiment following the manufacturer’s recommendations.

Animals were euthanized transferring them to an airtight chamber using 3% sevoflurane vapor inhalation with medical-grade oxygen as a carrier until total anesthesia, followed by exsanguination. Cardiac puncture was performed to collect cardiac blood until complete exsanguination, monitoring cessation of respiration and heartbeat, and the absence of reflexes as indicators of animal death.

### Characterization of the antibacterial effect of Saponin FR *in vitro*

To determine the antibacterial properties of Saponin FR fraction and QS-21, minimum inhibitory concentration (MIC) assays were performed on three bacterial strains with clinical significance. All antibacterial tests were carried out using fresh cultures in MHB, pelleted and suspended in aqueous NaCl 0.85% to a turbidity of 0.5 McFarland (1.5 × 10^8^ CFU/mL). Then a dilution was made to have in each well of a 96-well flat-bottomed plate 1 x10^6^ CFU/mL. Different amounts of Saponin FR or QS-21 were added to each well and were incubated at 37 °C, 150 rpm shaking for 48 h. Growth inhibition was recorded at 600 nm on an Epoch ELISA reader ELx800 (BioTek, VT, United States). Kanamycin at 3 μg/mL and a control without saponins was used. Antimicrobial activity was determined as the result of three independent experiments performed in triplicate.

### Characterization of the antifungal effect of Saponin FR *in vitro*

To determine the antifungal properties of Saponin FR, antifungal susceptibility testing was done by means of MIC assays, cultivating a *C*. *albicans* (ATCC 14053) in RPMI medium broth. Seeding of 1 × 10^4^ spores/well was performed in a 96-well flat-bottom plate, adding different concentrations of Saponin FR fraction and incubating at 37 °C, 150 rpm for up to 72 h. Fungal growth inhibition was determined on an Epoch ELISA reader (ELx800, BioTek, VT, United States) reading the absorbance at 600 nm. A control with saponin QS-21 was included as well as a control with no addition of saponins. Antifungal activity is given as the result of three independent experiments performed in triplicate.

### Virus propagation and titration

HeLa cells were grown until 80% of confluence in 75 cm^2^ culture flasks at 37 °C under controlled atmosphere - 5% CO_2_ and constant humidity. Cells were infected with adenovirus at a low multiplicity of infection (MOI) to ensure efficient replication. Once an 80%–90% cytopathic effect (CPE) was observed (usually 5–7 days), cells were freeze-thawed (three cycles) to release intracellular virions. Viral particles were recovered as previously described (Reed and Muench, 1938) and stored at −80 °C in glycerol. Tissue culture infectious dose at 50% (TCID50) assay was used to measure the infectious titers of VR-5. HeLa cells were grown to reach over 80% confluency in 96-well plates. Serial dilutions of VR-5 were added and were assayed by inoculation of 100 µL sample per well (10 wells were seeded per viral dilution) for 72 h, and CPE was observed by light microscopy. Cells were washed with PBS and fixed with 4% paraformaldehyde solution in PBS for 30 min. After discarding the para-formaldehyde solution, cells were stained by crystal violet for 1 h and then washed 3 times with PBS. The virus titer was calculated using the modified Kärber method (Lei et al., 2021), and cytopathogenicity was expressed as the tissue culture infectious dose at 50%/mL or TCID50/mL.

### Antiviral assays

The antiviral activity was determined by the quantitative carrier method under European standard EN 14476 (Reed and Muench, 1938; Tarka and Nitsch-Osuch, 2021). Briefly, HeLa cells were grown in DMEM medium containing 10% FBS and 1% penicillin–streptomycin in 25 cm^2^ culture flasks at 37 °C under controlled atmosphere 5% CO_2_ and constant humidity. Cell culture was subjected to infection at a MOI of 0.01 with VR-5 previously tittered as described above, under different non-cytotoxic concentrations of QS-21 and Saponin FR (2–250 μg/L), and the cell viability determined by MTT assay. Saponin FR and QS-21 were separately prepared in 2% DMSO and added just before infection, followed by incubation for 24 h at 37 °C in a humidified 5% CO_2_ atmosphere. Plates were incubated for a period corresponding to four cycles of multiplication (72 h) and examined daily by inverted light microscopy for the appearance of a CPE. MTT assay was used to determine the cell viability post infection according to the manufacturer’s instructions. Briefly, 10 μL of MTT reagent was added to each well and incubated for 4 h at 37 °C. The medium was removed, 100 μL DMSO were added and the absorbance at 570 nm was recorded using an Epoch ELISA reader ELx800 (BioTek, VT, United States). Controls of cell culture without virus infection (no infection control) and without saponin addition (infection control) were included. Antiviral activity is given as the result of at least three independent experiments performed in triplicate. Furthermore, the capacity to reduce viral duplication was evaluated by quantifying the % reduction of PFU/mL in the presence of QS-21 or Saponin FR, compared to the infection control.

### Characterization of the cytotoxic effect of Saponin FR *in vitro*

MTT assays were used to determine the inhibition of cancer cell proliferation by Saponin FR. Assays were performed with 3,000 cells/well of AGS and HeLa cell lines seeded into a 96-well plate in the respective medium and incubated during 24 h at 37 °C in a humidified 5% CO_2_ atmosphere. A stock of Saponin FR was prepared in 2% DMSO and serial dilutions were done in culture medium in a range between 0 and 50 mM; then, 10 µL were added to each well and were incubated by 24 h at 37 °C in a humidified 5% CO_2._ As positive control 10 µL of 100% ethanol were used, and negative control was only medium with 0.2% DMSO. MTT assay was performed according to the manufacturer’s instructions, as described above. The experiment was performed in triplicate.

## Results

### Saponin FR isolation and structural characterization

Saponin FR was isolated from a crude saponin water extract by semi-preparative reverse phase HPLC yielding a 99% pure compound. The UHPLC analysis rendered a single peak eluting at 3.7 min, as shown in the Supplementary Material file. Under the same chromatographic conditions, QS-21 saponin eluted at 3.9 min.

**FIGURE 1:**
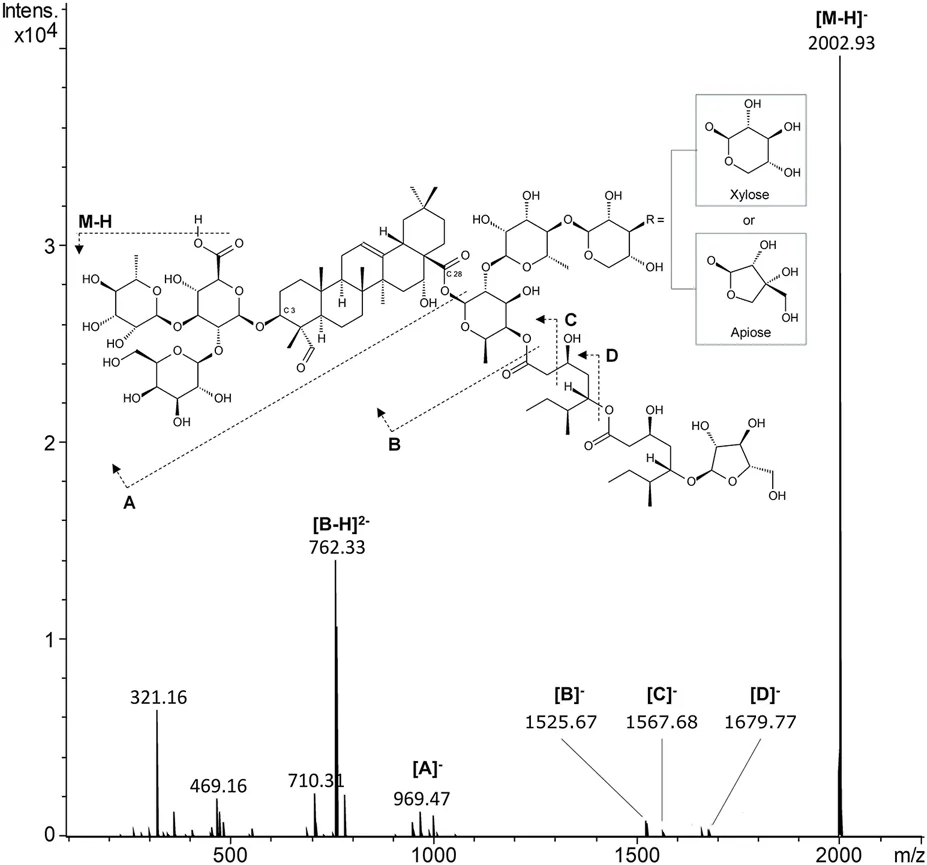
General structure of Saponin FR (molecular ion at [M-H]^-^ at m/z 2002.93), and the corresponding fragmentation pattern obtained in negative mode MS^2^ spectroscopy.

The negative mode ESI mass spectrum of Saponin FR (Figure 1) revealed the isotopic cluster of a quasi-molecular ion [M-H]ˉ. The most abundant ions in the cluster were found at m/z 2001.93 (monoisotopic ion) and 2002.93 (the most abundant ion containing a single ^13^C atom). The m/z value of the monoisotopic ion is 14 Th higher than the corresponding ion of the saponin QS-21 with formula C_92_H_148_O_46_ (Kite et al., 2004) suggesting the presence of an additional methyl group in Saponin FR. The above was confirmed by a cluster of single charged daughter ions dominated by ion A at m/z 969.47 previously reported to belong to the structure of several saponins from *Q. saponaria* with a rhamnose-substituted trisaccharide [*α*-l-rhamnopyranosyl-(1→3)-[*β*-d-galactopyranosyl-(1→2)]- *β*-d-glucuronopyranoside] attached to the C-3 hydroxyl of the aglycone (quillaic acid) (Kite et al., 2004). The corresponding trisaccharide in the ion A of QS-21 (at m/z 955.5) contains xylose instead of rhamnose, explaining the difference of 14 Th observed in the Saponin FR spectrum. The presence of glucuronic acid in the trisaccharide also explains the formation of the [M-H]ˉ ion by deprotonation of the carboxylic group at that sugar residue. Consistent with the presence of the rhamnosylated trisaccharide at C3 position is the formation of the daughter ions B, C and D at m/z 1525.67, 1567.68 and 1679.77 respectively (Figure 1). All these ions have m/z values shifted by 14 Th from the corresponding ions found in QS-21 (Wallace et al., 2022). An additional feature is the presence of a double charged ion cluster, dominated by ion [B-H]ˉ at m/z 762.33. Though the additional deprotonation site of this ion is not clear, the formation of double charged ions during negative ESI of some *Q. saponaria* saponins has been reported before Supplementary data from Jiang et al. (2019).

Based on the above fragmentation pattern, the reported similarity of *Quillaja* saponins (Kite et al., 2004) and the recently elucidated pathway for the biosynthesis of saponins in *Q. saponaria* (Martin et al., 2024; Reed et al., 2023), we conclude that Saponin FR differs from QS-21 only at the C-3 oligosaccharide. Therefore, Saponin FR should contain attached to the C-28 carboxylic group of quillaic acid any of the two tetrasaccharides found in QS-21: *β*-d-apiofuranosyl-(1→3)-*β*-d-xylopyranosyl-(1→4)-*α*-l-rhamnopyranosyl-(1→2)- *β*-d-fucopyranoside or *β*-d- xylopyranosyl-(1→3)-*β*-d-xylopyranosyl-(1→4)-*α*-l-rhamnopyranosyl-(1→2)- *β*-d-fucopyranoside. Any of these tetrasaccharides are further substituted at O4 position of the fucose residue with the 5-O-[5-O *α*-l-arabinofuranosyl-3,5-dihydroxy-6-methyl-octanoyl]-3,5-dihydroxy-6-methyl-octanoyl group. This structure (formula C_93_H_150_O_46_) is consistent with the fragmentation pattern shown in Figure 1, and the presence of rhamnose in the trisaccharide bound to the C3 position of the aglycone was confirmed by ^1^H, ^13^C, COSY and HSQC NMR (Supplementary Material file). Whether the single sugar substitution of Xyl by Rha impacts the biological activity of this novel saponin was further investigated.

### Immune response to Saponin FR *in vivo*

**FIGURE 2:**
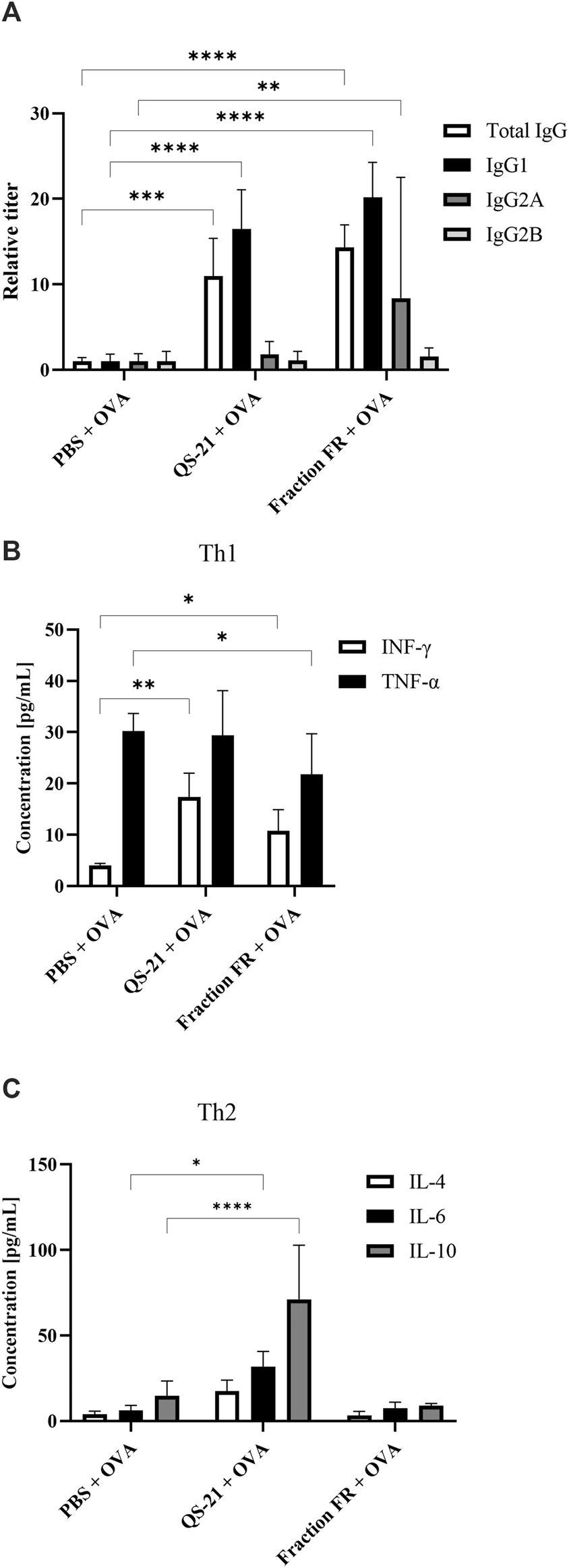
Comparison of: **(A)** *in vivo* immunoglobulin titers relative to control with ovalbumin antigen without saponins (total IgG and subtypes IgG1, IgG2A and IgG2B antibody production); **(B)** concentrations of secreted Th1 cytokines IFN-γ and TNF-α (middle panel); and **(C)** concentrations of secreted Th2 cytokines IL-4, IL-6 and IL-10 cytokine (bottom panel), all in sera from groups of animals (n = 7) treated with Saponin FR or QS-21. Mean relative titer values with the error expressed as standard deviation were compared to the negative control using Tukey’s multiple comparisons test with 95% confidence interval. *P < 0.05; **P < 0.01; ***P < 0.001; ****P < 0.0001.

The adjuvant effect of the Saponin FR fraction was evaluated in BALB/c mice. Saponin QS-21 was tested initially in several concentrations together with 50 µg OVA to determine a sub-lethal dose that was defined at 25 µg saponin. The analysis of the total IgGs titer (relative to the control with OVA and without saponins) shows that Saponin FR has comparable levels to the corresponding relative titer of QS-21. In both cases an approximate tenfold increase of total immunoglobulin titer was observed when compared to the control with no saponin addition (Figure 2, top panel). Looking at the IgG subtypes, both QS-21 and Saponins FR led to a predominant increase of IgG1 over the unadjuvanted control. Nevertheless, considerable levels of IgG2A were observed as well, particularly in the group treated with Saponin FR, pointing towards Th1/Th2 combined immune reactions. A former study showed that QS-21 induced significant levels of IgG2A and IgG2B. They had an IgG isotype profile similar to that observed in natural immunity arising from viral infections in mice, where IgG2A accounts for 65%–92% of total specific antibodies (Kensil et al., 1991). Linked to the previous observation, strong delayed hypersensitivity response and high IFN-γ splenocyte secretion, which is the main Th1-effector cytokine, are correlated to IgG2A in mice subcutaneously immunized with a fucose mannose ligand antigen of *Leishmania donovani* and QS-21 (Oliveira-Freitas et al., 2006). Following this rationale, levels of Th1 (IFN-γ and TNF-α) and Th2 (IL-4, IL-6 and IL-10) related-cytokines were evaluated in the final immune sera obtained for each treatment group (Figure 2, middle and bottom panels). The serum of animals treated with Saponin FR had only significant increases of IFN-γ. By contrast, animals treated with QS-21 showed significant increases of the serum concentrations of IFN-γ, IL-4, IL-6 and IL-10 compared to the control without saponins. Together these results indicate that Saponin FR primarily induces a Th1 response, whereas QS-21 presents a mixed Th1/Th2 immune response. A recent study reported that QS-21 or QS-18 as part of adjuvant phosphorylated hexa-acyl disaccharide and cholesterol liposomes delivered by a single injection in mice showed serum cytokine concentrations in the range of 1–30 pg/mL, close to the values presented here, but with no statistically significant differences to the untreated control. However, *in vitro* QS-18 incorporated liposome stimulation of mouse bone marrow dendritic cells generated a significant upregulation of Th1 immune response including pro-inflammatory cytokine TNF-α (Zhou et al., 2024).

### Antimicrobial effect of Saponin FR fraction *in vitro*

**FIGURE 3:**
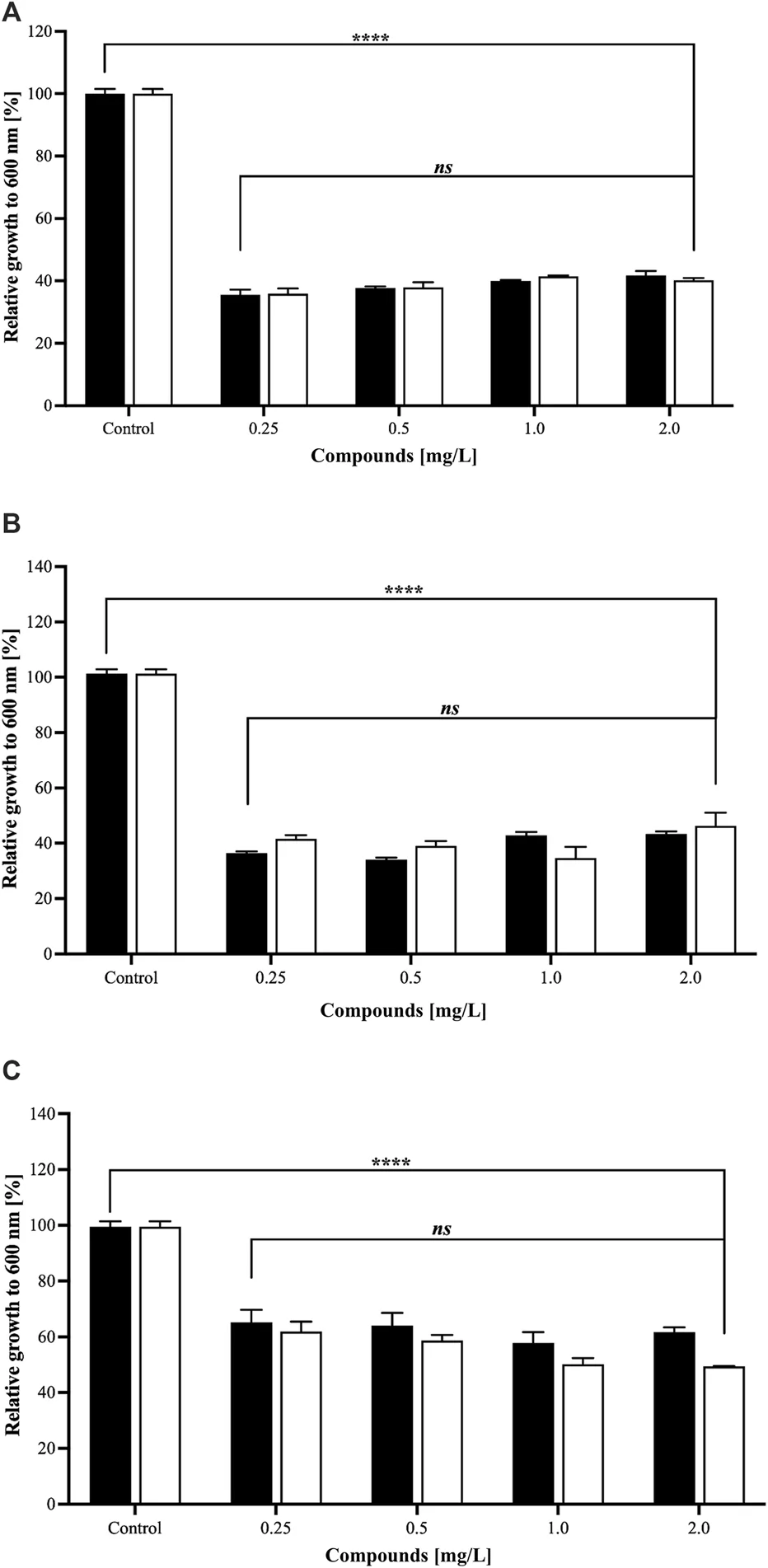
Antibacterial activity: Effect of Saponin FR (black bars) or QS-21 (white bars) on the growth of: **(A)** *Staphylococcus aureus* strain ATCC 25923; **(B)** a clinical isolate of *S. aureus* resistant to methicillin (MRSA), and; **(C)** *Klebsiella pneumoniae* strain ATCC 1705. Mean relative growth values with the error expressed as standard deviation were compared to control (bacterial cultures without saponins) using Tukey’s multiple comparisons test with 95% confidence interval. ns P > 0.05; ****P < 0.0001.

To determine the lowest concentration of Saponin FR and QS-21 that inhibit microbial growth, the MIC test was performed on cultures of *S. aureus* and a MRSA (clinical isolate) and *K. pneumoniae*. As observed in Figure 3, all tested strains were susceptible to Fraction FR and QS-21 in concentrations as low as 0.25 mg/L (0.12 µM) indicating a very high antibacterial effect of both saponins with 48 h of incubation in presence of the compound. The inhibitory growth effect was statistically significant across all tested strains, though it was stronger in *S. aureus* than in *K. pneumoniae* The MIC values are close to those of vancomycin for methicillin resistant *S. aureus* that are in the range from 0.3 to 5.5 µM (Moses et al., 2020). Previous studies have reported the antibacterial effect of saponin-rich *Quillaja* extracts. The MIC of a saponin-rich *Quillaja* extract was 100 mg/L against *S. aureus*, *S. typhimurium* and *E. coli*, while equivalent saponin-rich extracts of *Yucca schidigera* or *Cyamopsis tetragonoloba* (Guar) were more active against gram-positive *S. aureus* than gram-negative *E. coli* and *S. typhimurium*. In spite of our results and published evidence of antimicrobial effect of those compounds, not all saponins share the same properties; as an example, the saponin-rich soybean extract had no antibacterial activity against any bacteria at the concentrations tested by Hassan et al. (Hassan et al., 2010).

### Antifungal effect of Saponin FR *in vitro*

**FIGURE 4:**
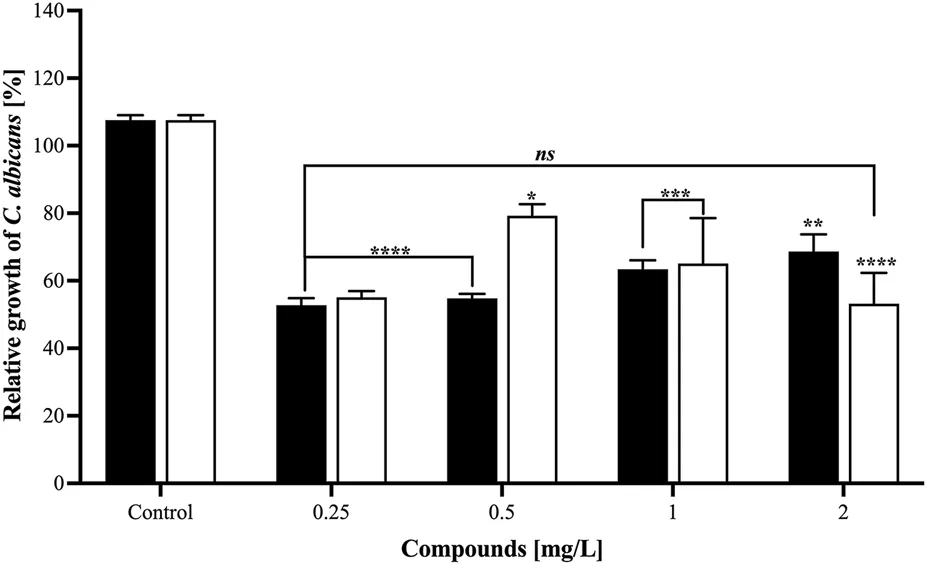
Antifungal activity: Effect of Saponin FR (black bars) or QS-21 (white bars) on the growth of *Candida albicans* clinical isolate. Mean relative growth values with the error expressed as standard deviation were compared to the control (yeast culture without saponins) using Tukey’s multiple comparisons test with 95% confidence interval. ns P > 0.05; *P < 0.05; **P < 0.01; ****P < 0.0001.

Abundant literature about saponin antifungal effects is available (Chapagain et al., 2007; Lu et al., 2023). Here we focus the analysis on the opportunistic fungal pathogen *C. albicans*, a common cause of human invasive infections where a limited number of therapeutic agents are available. The strain *C. albicans* was subjected to different concentrations of Saponin FR or QS-21, observing a statistically significant antifungal activity from a concentration of 0.25 mg/L (0.12 µM) as shown in Figure 4, where the viability of *C. albicans* decreases below a 60%. Previous studies have reported that *Q. saponaria* saponin-rich extracts (12% saponins in dry weight) have antifungal activity against phytopathogenic fungi at a concentration of 1 g/L (Chapagain et al., 2007). In the case of purified saponins, steroidal saponins such as the monodesmosidic from *Yucca schidigera* or saponin PS1 from *Paris polyphylla* has been reported MIC values of 7 or 1.8 µM against *C. albicans*, respectively (Miyakoshi et al., 2000; Chen et al., 2023). On the other hand, isolated triterpenic saponins, for example, Phytolaccoside B from *Phytolacca tetramera* Hauman or Anagallisin C from *Anagallis arvensis*, showed a wide range of MIC values from 188 to 0.9 µM against *C. albicans*, respectively, including clinical isolates in planktonic phase and biofilm forming isolates (Escalante et al., 2002; Soberón et al., 2017). Comparing these reported MIC values against *C. albicans* to those reported here for QS-21 and Saponin FR, there is a very significant potential for the use of the latter in clinical applications.

### Antiviral effect of Saponin FR *in vitro*

**FIGURE 5:**
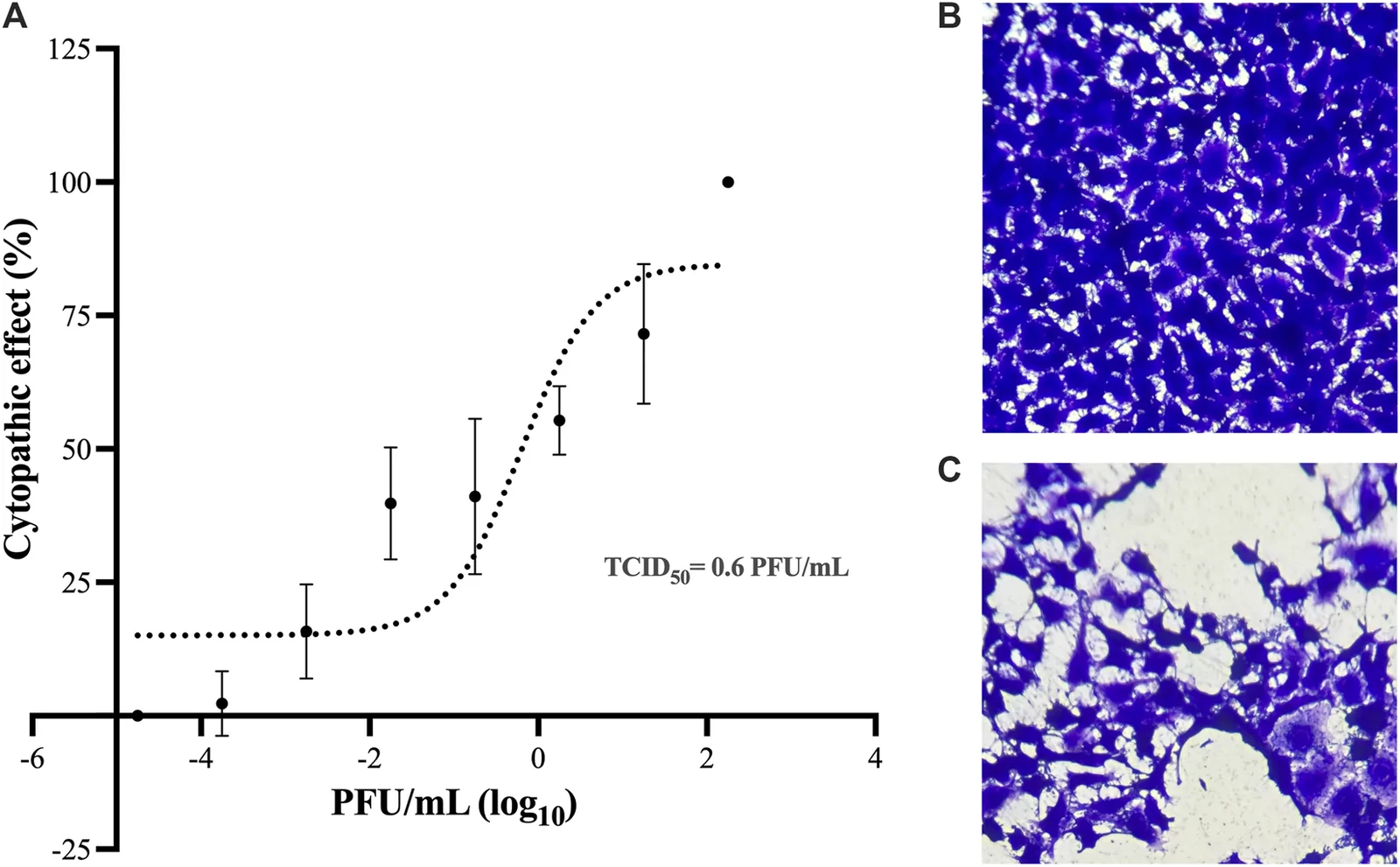
Determination of the infectious titer and cytopathic effect of human adenovirus (VR-5) on HeLa cell line: **(A)** Dose-response curve used to calculate TCID_50_ (3.16 × 10^5^ TCID_50_/mL); **(B)** cells not infected with the VR-5 virus, after crystal violet staining, and; **(C)** cytopathic effect of VR-5 on HeLa cells (dilution 10^–4^) after crystal violet staining.

**FIGURE 6:**
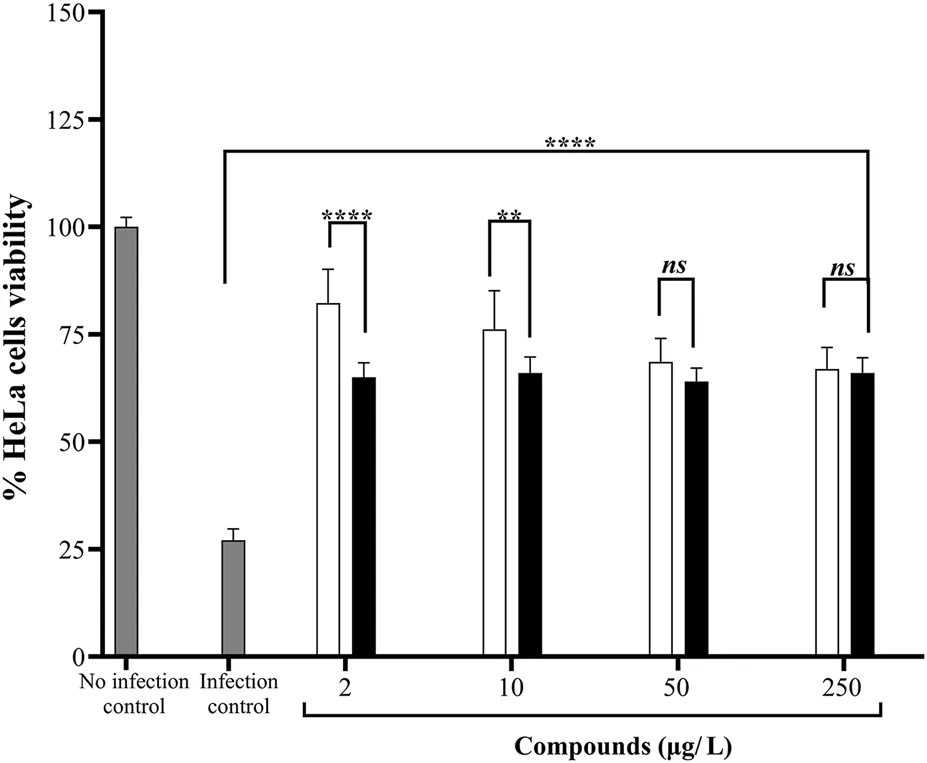
Antiviral effect of Saponin FR and QS-21 on HeLa cells. HeLa cell line subjected to infection with VR-5 (MOI of 0.01) in the presence of Saponin FR (black bars) or QS-21 (white bars). Comparison to HeLa cells cultured in medium (no infection control) and HeLa cells infected with VR-5 (MOI of 0.01) without saponins (infection control). Mean values of the percentage of cell viability are shown with error expressed as standard deviation for different concentrations of saponins. Treatments were compared between them and to controls using Kruskal–Wallis test with Bonferroni’s multiple comparisons at 95% confidence interval. ns P > 0.05; ****P < 0.0001.

To evaluate the antiviral properties of Saponin FR and QS-21, *in vitro* assays were performed using HeLa cells infected with VR-5 virus. Interestingly, VR-5 is a non-enveloped double-stranded DNA virus, which is stable outside the human body. Therefore, it is transmitted more broadly than enveloped ones. As shown in Figure 5, the no infection control demonstrates that saponins are non-toxic to HeLa cells in the concentration range employed in the study (2–250 μg/L). As expected, the infection control significantly reduced cell viability when infecting HeLa cells with VR-5 at the same viral multiplicity of infection (MOI), where the cytopathic effect was evaluated by microscopy and MTT assay as shown in Figure 6. Addition of Saponin FR or QS-21 just before infection showed that concentrations as low as 2 μg/L can significantly reduce VR-5 infectivity. Previous studies have shown that *Q. saponaria* saponins have antiviral activity against enveloped and non-enveloped viruses at 100 μg/L (Roner et al., 2007). The antiviral activity of a natural triterpenic monodesmosidic saponin isolated from *Anagallis arvensis* was 800 μg/L for the non-enveloped type 2 poliovirus (Amoros et al., 1987). This value is several orders of magnitude higher than the values observed for Saponin FR and QS-21. However, the large value previously observed for *Quillaja* saponins was obtained with extracts with a complex saponin composition instead of highly purified fractions such as those reported here. Additional previous studies with *Q. saponaria* saponin mixtures have shown a direct antiviral activity against enveloped and non-enveloped viruses at 100 and 1000 μg/L, respectively, as well as a transient resistance to infection for a period of 16 h after treatment with 100 μg/L for non-enveloped reovirus and rotavirus (Roner et al., 2010).

### Antitumoral effect of Saponin FR fraction *in vitro*

**FIGURE 7:**
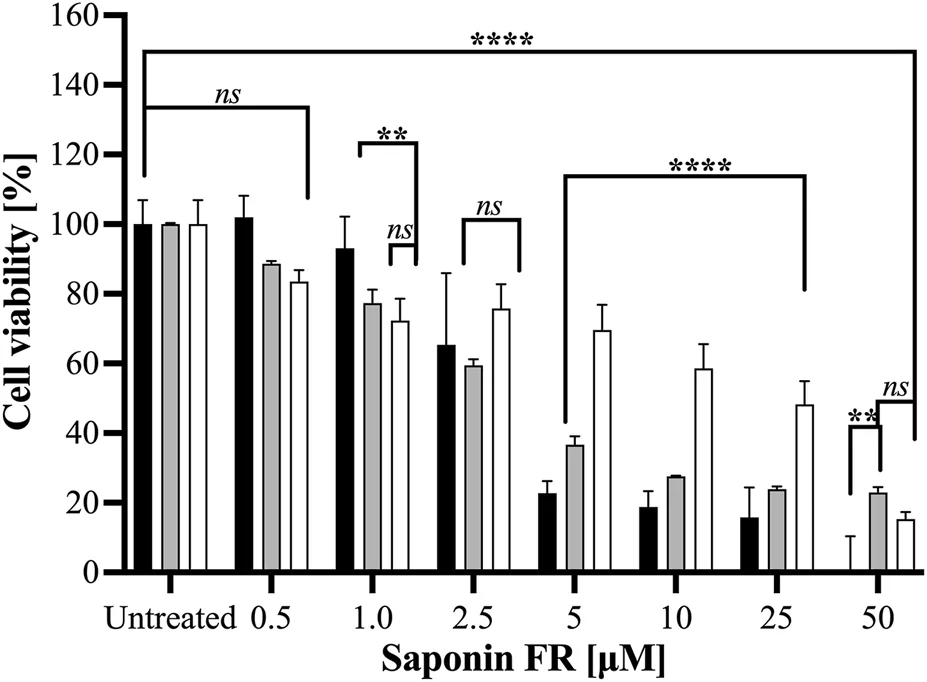
Effect of Saponin FR on cell viability by MTT assay. Cells were incubated with different concentrations of Saponin FR for 24 h. Gastric adenocarcinoma cell line AGS (black), cervix adenocarcinoma cell line HeLa (gray) and healthy control human gastric epithelial cell line GES-1 (white). Data expressed as means ± DS of five independent experiments were compared with untreated cells using two-way ANOVA with Tukey’s multiple comparisons at 95% confidence interval. ns P > 0.05; *P < 0.05; **P < 0.01; ***P < 0.001; ****P < 0.0001.

An important aspect of the isolation and characterization of new natural compounds lies in their pharmacological activity. In this context, new molecules able to selectively target uncontrolled proliferative cells without affecting normal cells are of outmost importance to develop better and new cancer treatments. In this study we determine cancer cell proliferation inhibition with different concentrations of Saponin FR on two cell lines from gastric (AGS) and cervix (HeLa) adenocarcinoma. The cytotoxic effect of these triterpenic saponins is shown in Figure 7, where Saponin FR was cytotoxic to AGS and HeLa tumor lines in a dose dependent way (IC_50_ values of 2.9 and 2.6 μM, respectively). The exposure of both cancer cell lines to Saponin FR in a range from 1.0 µM to 25 µM resulted in a dose-dependent cell viability compared with untreated cells and GES-1 normal cells (P < 0.0001) that showed a significantly higher tolerance (IC_50_ 193.4 µM).

This result is consistent with published results of cytotoxic activity of saponins. Saponin QS-21 has a cytotoxic effect on human gastric cancer cells such as SNU1 and KATO III with IC_50_ values of 7 µM for both cell lines, 3 times lower than previously studied with triterpenoid saponin extracted from the roots of *Adenophora triphylla* var. japonica (Guzman et al., 2020; Lu et al., 2015). Other examples of antiproliferative effect of triterpenic saponins can be mentioned; for instance the monodesmosidic saponins Asperosaponin C from *Dipsacus asper* with an IC_50_ of 30 µM against stomach cancer cell line BGC-823 (Ji et al., 2012), or Erythroside C isolated from *Erythrophleum fordii* Oliver, that possess cytotoxic effect against human lung cancer cell line PC9 with IC_50_ at 13 µM (Chen et al., 2020). Remarkably, two triterpenic monodesmosidic saponins isolated from *Camellia oleifera* Abel, have antiproliferative activity with an IC_50_ between 1 and 18 µM against several cancer cell lines, including non-small cell lung adenocarcinoma, ovarian cancer cells, skin melanoma and colon cancer cells, and the cytotoxic activity of these saponins against colon cancer cells was greater than the anticancer drug mitoxantrone (IC_50_ 4 µM compared to 1 µM) (Zhou et al., 2014), highlighting the relevance of purified triterpenic saponins as antiproliferative agents for cancer treatment.

## Discussion

A current major need in the frame of the development of new and better vaccine technology with more safe and effective immune response induction and focusing on the current high purity subunit vaccines and their effect over vulnerable social groups, require the discovery and characterization of new vaccine adjuvants able to bring a strong and prolonged immunity both humoral and cellular. In this sense the most studied *Quillaja* saponin QS-21 has been underlined to possess a collection of immunostimulant activities including both immune responses Th1, against intracellular pathogens, and Th2 to purge extracellular parasites and bacterial infections (Marciani, 2018). However, interest has focused only on the enriched saponin fractions among the standard *Quillaja* mixture including QS-21, and many studies have characterized the different effects of complete *Quillaja* extracts that have a plethora of pharmacological activities comprising additional antitumoral, antimicrobial, antiviral and antifungal properties. Here we draw attention to the isolation and characterization of Saponin FR, a rhamnosylated triterpenic saponin of *Q. saponaria* with potent adjuvant, antimicrobial, antiproliferative and antiviral effects. The novel saponin structurally differs from QS-21 by a single sugar (xylose is replaced by rhamnose in the trisaccharide attached to the C-3 of the aglycone core). This difference impacts its immunostimulant activities, being Saponin FR more prone towards the induction of a Th1 response instead of a mixed Th1/Th2 as in the case of QS-21. Previously, N-acylations in the C-3 of *Quillaja* saponin derivatives that increase their hydrophobicity, have shown different immune modulatory properties depending on their conformation and micellar state, favoring a Th1 immune response (Marciani, 2022). Regarding this, a successful vaccine development depends mainly on the stimulation of an appropriate set of immune effectors against the specific pathogen. Ideally, both Th1 and Th2 immune responses should be activated, the first to endorse a cell-mediated response, and the second to promote a humoral response. Here, Saponin FR is now available to become part of new adjuvant formulations as the component to specifically enhance Th1 immune response to deal with intracellular pathogens or to promote mixed Th1/Th2 induction in mixtures with other immunostimulant compounds.

Concerning the different antibacterial effects of *Quillaja* saponins on *S. aureus* and *K. pneumoniae*, previous reports attribute this to the degradation of saponins by non-specific glycosidases capable of hydrolyzing the glycosidic bonds of saponins, and that are produced in higher concentrations by Gram-negative than Gram-positive bacteria (Sen et al., 1998). Recent reports show that the triterpenic saponin-rich fractions of *Calliandra umbellifera* and *Sarcomphalus joazeiro* showed MIC values equal or higher than 1024 mg/L for multidrug resistant strains of *E. coli*, *S. aureus* and *P. aeruginosa*, several orders of magnitude above the observed MIC for FR fraction purified and characterized here. Interestingly the combination of these saponin-rich fractions with antibiotics showed variable effects, including a gentamicin enhancement mainly against Gram-negative strains, and a decreased effect of ampicillin against *S. aureus* (Costa et al., 2024; da Silva et al., 2024).

With respect to antiviral properties of Saponin FR, very interesting results were observed since a very low concentration (2 μg/L) is able to reduce HeLa cells infection by non-enveloped virus VR-5 challenge, being 400 times lower than previously reported for *Quillaja* saponin mixtures in the presence of different non-enveloped viruses (Roner et al., 2007; Roner et al., 2010). Available reports for selective antiviral activity of purified triterpenic saponins isolated from *A. arvensis* showed activity against enveloped type 1 herpes simplex virus (HSV-1) and non-enveloped type 2 poliovirus, but not against non-enveloped human adenovirus 6, and enveloped vaccinia and vesicular stomatitis viruses (Amoros et al., 1987), indicating a very specific antiviral effect possibly associated to modifications of the cell surface preventing specific virus attaching and/or interference with the specific virus replicative cycle within the cell. As with antibiotics, antiviral comparisons are performed in molar concentration terms. In this sense, Herpes simplex virus 1 (HSV-1) antiviral activity of two triterpenoid saponins from *Sargentodoxa cuneata* and *Thinouia coriacea* were 25 and 2.7 µM, respectively (Simões et al., 1999). Here we report that *Q. saponaria* Saponin FR has virucidal effect at a concentration of 1 nM, below the 36 nM concentration reported for the commercial adenovirus antiviral compound Cidofovir (De Clercq, 2002), therefore being an interesting candidate for new commercial antiviral formulations.

As mentioned before, perhaps the antifungal activity of saponins is the most extensively studied among the antimicrobial properties of these secondary metabolites.

Here we observed that Fraction FR possess strong activity against a *C. albicans* strain considering the low concentration value able to significantly inhibit its growth (0.13 µM). Comparing this value with the one observed for the standard antifungal compound fluconazole against similar *C. albicans* strains, the synthetic triazole antifungal agent has MIC values around 0.8 µM (Pfaller et al., 2006), indicating a new potential use of these saponins as part of antifungal formulations for clinical use. This is of outmost importance considering that currently there is a reduced number of therapeutic antifungal agents, and a decrease in the activity of the available ones due to an increase of the resistance mechanisms in these microorganisms, particularly the ability to form biofilms which is a characteristic of most candidiasis related infections. Interestingly, the use of combinations of synthetic antifungals and saponins have shown synergistic effects, for example, the combination of a semi-synthetic analogue of *Panax stipulcanatus* monodesmosidic triterpenic saponins with fluconazole, showed an antifungal activity against fluconazole-resistant *C. albicans* (Yin et al., 2022). Finally, in line with prior findings for QS-21 (Guzmán et al., 2020), Saponin FR demonstrated cytotoxic activity against two adenocarcinoma cell lines (AGS and HeLa), with IC_50_ values under 3 µM and no damage to healthy gastric cells (GES-1; Figure 7). Comparing these results with previously reported ones, isolated triterpenic monodesmosidic saponins from the sea cucumber *Cercodemas anceps* have shown strong cytotoxic effect on Hep-G2 (hepatoma cancer), KB (epidermoid carcinoma), LNCaP (prostate cancer), MCF7 (breast cancer) and SK-Mel2 (melanoma) with IC_50_ values in the range of 0.03–7 μM, being stronger than the positive control ellipticine (Cuong et al., 2015). Likewise, triterpenic bidesmosidic saponin isolated from *Actinostemma lobatum* Maxim showed high antiproliferative activity against a breast cancer cell line with an IC_50_ of 8 µM below the 12 µM value for cis-Platinum (Cao et al., 2015), showing that the antiproliferative effect of purified triterpenic saponins is equal and even stronger than currently used anticancer drugs being selective against tumoral cells. Regarding the cytotoxic mechanism associated with triterpenic saponins, for Ardisiacrispin B isolated from *Ardisia kivuensis* Taton, that has an antiproliferative activity against multi-drug resistant leukemia cell subline CEM/ADR5000 with an IC_50_ more than 25 times lower than the anticancer drug doxorubicin (4 vs. 123 µM), its cytotoxicity was associated to the induction of apoptosis via increase in intracellular concentrations of radical oxygen species, activation of initiator and effector caspases, and alteration of mitochondrial membrane potential (Mbaveng et al., 2018).

Overall, the novel *Quillaja saponaria* Molina rhamnosylated triterpenic Saponin FR, possesses an extraordinary potential to be applied in new vaccine adjuvant formulations, as well as to become part of different antimicrobial, antiviral and antitumoral clinical applications.

## Data Availability

The raw data supporting the conclusions of this article will be made available by the authors, without undue reservation.

## References

[B1] Amoros M. Fauconnier B. Girre R. L. (1987). *In vitro* antiviral activity of a saponin from *Anagallis arvensis*, primulaceae, against herpes simplex virus and poliovirus. Antivir. Res. 8 (8), 13–25. 10.1016/0166-3542(87)90084-2 2825589

[B2] Berlowska J. Dudkiewicz M. Kregiel D. Czyzowska A. Witonska I. (2015). Cell lysis induced by membrane-damaging detergent saponins from *Quillaja saponaria* . Enzyme Microb. Technol. 75-76, 44–48. 10.1016/j.enzmictec.2015.04.007 26047915

[B3] Cao J. Li W. Tang Y. Zhang X. Li W. Zhao Y. (2015). Three new triterpene saponins from *Actinostemma lobatum* MAXIM and their cytotoxicity *in vitro* . Phytochem. Lett. 11, 301–305. 10.1016/j.phytol.2015.01.016

[B4] Chapagain B. P. Wiesman Z. Tsror L. (2007). *In vitro* study of the antifungal activity of saponin-rich extracts against prevalent phytopathogenic fungi. Ind. Crops Prod. 26, 109–115. 10.1016/j.indcrop.2007.02.005

[B5] Chavali S. R. Francis T. Campbell J. B. (1987). An *in vitro* study of immunomodulatory effects of some saponins. Int. J. Immunopharmacol. 9, 675–683. 10.1016/0192-0561(87)90038-5 3500923

[B6] Chen Z. P. Guo L. B. He J. Xu J. K. Li Y. N. Huang X. Y. (2020). Triterpene saponins from the seeds of *Erythrophleum fordii* and their cytotoxic activities. Phytochemistry 177, 112428. 10.1016/j.phytochem.2020.112428 32535346

[B7] Chen Y. Yan Q. Ji Y. Bai X. Li D. Mu R. (2023). Unraveling the serial glycosylation in the biosynthesis of steroidal saponins in the medicinal plant *Paris polyphylla* and their antifungal action. Acta Pharm. Sin. B 13, 4638–4654. 10.1016/j.apsb.2023.05.033 37969733 PMC10638507

[B8] Costa M. S. da Silva A. R. Araújo N. J. Paulo C. L. Ribeiro P. R. Tavares J. F. (2024). UPLC–QTOF-MS/MS analysis of saponin-enriched fractions from *Calliandra umbellifera* benth and evaluation of antibacterial activity against multidrug-resistant bacteria. Phytochem. Lett. 59, 64–68. 10.1016/j.phytol.2023.11.002

[B9] Cuong N. X. Vien le T. Hanh T. T. Thao N. P. Thao do T. Thanh N. V. (2015). Cytotoxic triterpene saponins from *Cercodemas anceps* . Bioorg Med. Chem. Lett. 25, 3151–3156. 10.1016/j.bmcl.2015.06.005 26099533

[B10] da Silva A. R. Costa M. S. Araújo N. S. Alencar G. Ribeiro P. R. Tavares J. F. (2024). UPLC-QTOF-MS/MS characterization of a fraction enriched in saponins from *Sarcomphalus joazeiro* species and evaluation of the antibacterial activity against multidrug-resistant bacteria. S. Af. J. Bot. 165, 324–330. 10.1016/j.sajb.2024.01.007

[B11] Dalsgaard A. (1978). Study of the isolation and characterization of the saponin Quil A. Evaluation of its adjuvant activity, with a special reference to the application in the vaccination of cattle against foot-and-mouth disease. Acta Vet. Scand. Suppl. 69, 7–40. 102135

[B12] De C. E. (2002). Cidofovir in the treatment of poxvirus infections. Antivir. Res. 55, 1–13. 10.1016/s0166-3542(02)00008-6 12076747 PMC9533828

[B13] Escalante A. M. Santecchia C. B. López S. N. Gattuso M. A. Gutiérrez Ravelo A. Delle Monache F. (2002). Isolation of antifungal saponins from *Phytolacca tetramera*, an Argentinean species in critic risk. J. Ethnopharmacol. 82, 29–34. 10.1016/s0378-8741(02)00145-9 12169402

[B14] Fleck J. D. Betti A. H. da Silva F. P. Troian E. A. Olivaro C. Ferreira F. (2019). Saponins from *Quillaja saponaria* and *Quillaja brasiliensis*: particular chemical characteristics and biological activities. Molecules 24, 171. 10.3390/molecules24010171 30621160 PMC6337100

[B15] Guzmán L. Villalón K. Marchant M. J. Tarnok M. E. Cárdenas P. Aquea G. (2020). *In vitro* evaluation and molecular docking of QS-21 and quillaic acid from *Quillaja saponaria* Molina as gastric cancer agents. Sci. Rep. 10, 10534. 10.1038/s41598-020-67442-3 32601436 PMC7324585

[B16] Hassan S. M. Byrd J. A. Cartwright A. L. Bailey C. A. (2010). Hemolytic and antimicrobial activities differ among saponin-rich extracts from guar, quillaja, yucca, and soybean. Appl. Biochem. Biotechnol. 162, 1008–1017. 10.1007/s12010-009-8838-y 19915999

[B17] Hostettmann K. A. Marston A. (1995). Saponins. Cambridge: Cambridge University Press. 10.1017/CBO9780511565113

[B18] Ji D. Wu Y. Zhang B. Zhang C. F. Yang Z. L. (2012). Triterpene saponins from the roots of *Dipsacus asper* and their protective effects against the Aβ(25-35) induced cytotoxicity in PC12 cells. Fitoterapia 83, 843–848. 10.1016/j.fitote.2012.03.004 22430115

[B19] Jiang X. Strobel B. W. Cedergreen N. Cao Y. Hansen H. C. B. (2019). Stability of saponin biopesticides: hydrolysis in aqueous solutions and lake waters. Environ. Sci. Process Impacts 21, 1204–1214. 10.1039/c9em00012g 31241099

[B20] Kaper J. B. Nataro J. P. Mobley H. L. (2004). Pathogenic Escherichia coli. Nat. Rev. Microbiol. 2, 123–140. 10.1038/nrmicro818 15040260

[B21] Kensil C. A. Marciani D. J. (1991). “Saponin adjuvant,” in US Patent N° 5,057, 540.

[B22] Kensil C. R. Patel U. Lennick M. Marciani D. (1991). Separation and characterization of saponins with adjuvant activity from *Quillaja saponaria* Molina cortex. J. Immunol. 146, 431–437. 10.4049/jimmunol.146.2.431 1987271

[B23] Kite G. C. Howes M. J. Simmonds M. S. (2004). Metabolomic analysis of saponins in crude extracts of *Quillaja saponaria* by liquid chromatography/mass spectrometry for product authentication. Rapid Commun. Mass Spectrom. 18, 2859–2870. 10.1002/rcm.1698 15517552

[B24] Lei C. Yang J. Hu J. Sun X. (2021). On the calculation of TCID_50_ for quantitation of virus infectivity. Virol. Sin. 36, 141–144. 10.1007/s12250-020-00230-5 32458296 PMC7973348

[B25] Lu Y. Van D. Deibert L. Bishop G. Balsevich J. (2015). Antiproliferative quillaic acid and gypsogenin saponins from *Saponaria officinalis* L. roots. Phytochemistry 113, 108–120. 10.1016/j.phytochem.2014.11.021 25534953

[B26] Lu L. Min-ping W. Hang Y. Yun-fei X. Ya-hui G. Yu-liang C. (2023). Antifungal activity of *Sapindus* saponins against *Candida albicans*: interruption of biofilm formation. J. Herb. Med. 42, 100776. 10.1016/j.hermed.2023.100776

[B27] Magedans Y. V. Yendo A. C. Costa F. Gosmann G. Fett-Neto A. G. (2019). Foamy matters: an update on Quillaja saponins and their use as immunoadjuvants. Future Med. Chem. 11:1485–1499. 10.4155/fmc-2018-0438 31304830

[B28] Marciani D. J. (2018). Elucidating the mechanisms of action of saponin-derived adjuvants. Trends Pharmacol. Sci. 39, 573–585. 10.1016/j.tips.2018.03.005 29655658

[B29] Marciani D. J. (2022). Effects of N-acylation on the immune adjuvanticity of analogs of the *Quillaja* saponins derivative GPI-0100. Vaccine 40, 4169–4173. 10.1016/j.vaccine.2022.05.084 35688726

[B30] Martin L. B. B. Kikuchi S. Rejzek M. Owen C. Reed J. Orme A. (2024). Complete biosynthesis of the potent vaccine adjuvant QS-21. Nat. Chem. Biol. 20, 493–502. 10.1038/s41589-023-01538-5 38278997 PMC10972754

[B31] Mbaveng A. T. Ndontsa B. L. Kuete V. Nguekeu Y. M. M. Çelik İ. Mbouangouere R. (2018). A naturally occurring triterpene saponin ardisiacrispin B displayed cytotoxic effects in multi-factorial drug resistant cancer cells via ferroptotic and apoptotic cell death. Phytomedicine 43, 78–85. 10.1016/j.phymed.2018.03.035 29747757

[B32] Miyakoshi M. Tamura Y. Masuda H. Mizutani K. Tanaka O. Ikeda T. (2000). Antiyeast steroidal saponins from *Yucca schidigera* (Mohave yucca), a new anti-food-deteriorating agent. J. Nat. Prod. 63, 332–338. 10.1021/np9904354 10757713

[B33] Montenegro G. Peña R. C. Timmermann B. N. (2001). La corteza de quillay (*Quillaja saponaria* Mol.), un recurso de la faramacopea internacional. Rev. Acad. Colomb. Cienc. 25, 421–427. 10.18257/raccefyn.25(96).2001.2811

[B34] Moses V. K. Kandi V. Rao S. K. D. (2020). Minimum inhibitory concentrations of vancomycin and daptomycin against methicillin-resistant *Staphylococcus aureus* isolated from various clinical specimens: a study from South India. Cureus 12, e6749. 10.7759/cureus.6749 32140317 PMC7039364

[B35] Oliveira-Freitas E. Casas C. P. Borja-Cabrera G. P. Santos F. N. Nico D. Souza L. O. (2006). Acylated and deacylated saponins of *Quillaja saponaria* mixture as adjuvants for the FML-vaccine against visceral leishmaniasis. Vaccine 24, 3909–3920. 10.1016/j.vaccine.2006.02.034 16556475

[B36] Padilla L. González J. Otero R. (2022). Production of biomass in ultra high density plantations. U. S. Patent N° 11,254,699 B2.

[B37] Pfaller M. A. Diekema D. J. Sheehan D. J. (2006). Interpretive breakpoints for fluconazole and Candida revisited: a blueprint for the future of antifungal susceptibility testing. Clin. Microbiol. Rev. 19, 435–447. 10.1128/CMR.19.2.435-447.2006 16614256 PMC1471993

[B38] Reed L. J. Muench H. (1938). A simple method of estimating fifty per cent endpoints. Am. J. Epidemiol. 27, 493–497. 10.1093/oxfordjournals.aje.a118408

[B39] Reed J. Orme A. El-Demerdash A. Owen C. Martin L. B. B. Misra R. C. (2023). Elucidation of the pathway for biosynthesis of saponin adjuvants from the soapbark tree. Science 379, 1252–1264. 10.1126/science.adf3727 36952412

[B40] Roner M. R. Sprayberry J. Spinks M. Dhanji S. (2007). Antiviral activity obtained from aqueous extracts of the Chilean soapbark tree (*Quillaja saponaria* Molina). J. Gen. Virol. 88, 275–285. 10.1099/vir.0.82321-0 17170461

[B41] Roner M. R. Tam K. I. Kiesling-Barrager M. (2010). Prevention of rotavirus infections *in vitro* with aqueous extracts of *Quillaja saponaria* Molina. Future Med. Chem. 2, 1083–1097. 10.4155/fmc.10.206 20725585 PMC2921663

[B42] Schwarz T. F. Hwang S. J. Ylisastigui P. Liu C. S. Takazawa K. Yono M. (2024). Immunogenicity and safety following 1 dose of AS01E-adjuvanted respiratory syncytial virus prefusion f protein vaccine in older adults: a phase 3 trial. J. Infect. Dis. 230, e102–e110. 10.1093/infdis/jiad546 39052726 PMC11272088

[B43] Sen S. Makkar H. P. Muetzel S. Becker K. (1998). Effect of *Quillaja saponaria* saponins and *Yucca schidigera* plant extract on growth of *Escherichia coli* . Lett. Appl. Microbiol. 27, 35–38. 10.1046/j.1472-765x.1998.00379.x 9722995

[B44] Shinde V. Cho I. Plested J. S. Agrawal S. Fiske J. Cai R. (2022). Comparison of the safety and immunogenicity of a novel Matrix-M-adjuvanted nanoparticle influenza vaccine with a quadrivalent seasonal influenza vaccine in older adults: a phase 3 randomised controlled trial. Lancet Infect. Dis. 22, 73–84. 10.1016/S1473-3099(21)00192-4 34563277

[B45] Simões C. M. Amoros M. Girre L. (1999). Mechanism of antiviral activity of triterpenoid saponins. Phytother. Res. 13, 323–328. 10.1002/(SICI)1099-1573(199906)13:4<323:AID-PTR448>3.0.CO;2-C 10404540

[B46] Simon A. K. Hollander G. A. McMichael A. (2015). Evolution of the immune system in humans from infancy to old age. Proc. Biol. Sci. 282, 20143085. 10.1098/rspb.2014.3085 26702035 PMC4707740

[B47] Singh G. Song S. Choi E. Lee P. B. Nahm F. S. (2020). Recombinant zoster vaccine (Shingrix®): a new option for the prevention of herpes zoster and postherpetic neuralgia. Korean J. Pain 33, 201–207. 10.3344/kjp.2020.33.3.201 32606264 PMC7336348

[B48] Snapper C. M. Paul W. E. (1987). Interferon-gamma and B cell stimulatory factor-1 reciprocally regulate Ig isotype production. Science. 236, 944–947. 10.1126/science.3107127 3107127

[B49] Soberón J. R. Sgariglia M. A. Pastoriza A. C. Soruco E. M. Jäger S. N. Labadie G. R. (2017). Antifungal activity and cytotoxicity of extracts and triterpenoid saponins obtained from the aerial parts of *Anagallis arvensis* L. J. Ethnopharmacol. 203, 233–240. 10.1016/j.jep.2017.03.056 28389355

[B50] Sun H. X. Xie Y. Ye Y. P. (2009). Advances in saponin-based adjuvants. Vaccine 27, 1787–1796. 10.1016/j.vaccine.2009.01.091 19208455

[B51] Syed Y. Y. (2022). RTS,S/AS01 malaria vaccine (Mosquirix®): a profile of its use. Drugs Ther. Perspect. 38, 373–381. 10.1007/s40267-022-00937-3 36093265 PMC9449949

[B52] Tarka P. Nitsch-Osuch A. (2021). Evaluating the virucidal activity of disinfectants according to European Union standards. Viruses 13, 534. 10.3390/v13040534 33804814 PMC8063834

[B53] Underwood E. Dunkle L. M. Madhi S. A. Gay C. L. Heath P. T. Kotloff K. L. (2023). Safety, efficacy, and immunogenicity of the NVX-CoV2373 vaccine. Expert Rev. Vaccines 22, 501–517. 10.1080/14760584.2023.2218913 37246757

[B54] Wallace F. Fontana C. Ferreira F. Olivaro C. (2022). Structure elucidation of triterpenoid saponins found in an immunoadjuvant preparation of *Quillaja brasiliensis* using mass spectrometry and ^1^H and ^13^C NMR spectroscopy. Molecules 27, 2402. 10.3390/molecules27082402 35458600 PMC9024837

[B55] Wang Z. B. Xu J. (2020). Better adjuvants for better vaccines: progress in adjuvant delivery systems, modifications, and adjuvant-antigen codelivery. Vaccines (Basel) 8, 128. 10.3390/vaccines8010128 32183209 PMC7157724

[B56] Yin S. Li L. Su L. Li H. Zhao Y. Wu Y. (2022). Synthesis and *in vitro* synergistic antifungal activity of analogues of *Panax stipulcanatus* saponin against fluconazole-resistant *Candida albicans* . Carbohydr. Res. 517, 108575. 10.1016/j.carres.2022.108575 35552063

[B57] Zhou H. Wang C. Z. Ye J. Z. Chen H. X. (2014). New triterpene saponins from the seed cake of *Camellia oleifera* and their cytotoxic activity. Phytochem. Lett. 8, 46–51. 10.1016/j.phytol.2014.01.006

[B58] Zhou S. Song Y. Nilam A. Luo Y. Huang W. C. Long M. D. (2024). The predominant *Quillaja saponaria* fraction, QS-18, is safe and effective when formulated in a liposomal murine cancer peptide vaccine. J. Control Release 369, 687–695. 10.1016/j.jconrel.2024.04.002 38575073

