# Supplementary material for: The characterization of a rhamnosylated triterpenic saponin from Quillaja saponaria Mol. reveals novel adjuvant, antibacterial, antiviral, antifungal and antiproliferative properties
Source: Front Pharmacol. 2026 Aug 26;17:1856067. doi: 10.3389/fphar.2026.1856067 (PMC13555342; doi:10.3389/fphar.2026.1856067)
Supplement: Supplementary file 1 [file DataSheet1.pdf]

The characterization of a rhamnosylated triterpenic saponin from *Quillaja saponaria* Mol. reveals novel adjuvant, antibacterial, antiviral, antifungal and antiproliferative properties.

Roberto Bobadilla-Fazzini[1], María José Marchant[2], Elisabeth Gromahan[3], Leda Guzmán[2]* & Leandro Padilla[1]*

[1]Desert King Chile, Valparaíso 3100000, Chile.

[2]Laboratorio de Química Biológica, Instituto de Química, Facultad de Ciencias, Pontificia Universidad Católica de Valparaíso, Valparaíso 23732223, Chile

[3] Department of Microbiology, Faculty of Life Sciences and Technology, Berlin University of Applied Sciences, 13347 Berlin, Germany

Spectroscopic characterization of Saponin FR and QS-21 by nuclear magnetic resonance (^{1}H and ^{13}C NMR).

Quillaja saponaria is an abundant source of triterpenic saponins. These compounds are bidesmosidic glycosides of quillaic acid with 3-O and 28-O bonded glycosyl moieties (Van Setten *et al*., 1995); the sugar substituents present in *Q. saponaria* saponins may be α-L-rhamnose, β-D-galactose, β-D-xylose, β-D-glucuronic acid, β-D-fucose, β-D-glucose, β-D-apiose and/or α-L-arabinose (Nord & Kenne 1999, 2000). According to Van Setten *et al*. (1995) there are two series of saponins based on the composition of their 3-O substituent: xylosylated saponins (containing glucuronic acid, galactose and one xylose) and rhamnosylated saponins (containing glucuronic acid, galactose and one rhamnose). The biochemical basis of the occurrence of both series was provided by the recently published biosynthetic pathway of *Q. saponaria* (Martin *et al*., 2024; Reed *et al*., 2023).

QS-21 is a chromatographic fraction of saponins containing the xylosylated trisaccharide 3-O-[β-D-xylopyranosyl-(1→3)-[β-D-galactopyranosyl-(1→2)]-β-D-glucuronopyranoside] bonded to position 3-O of the quillaic acid aglycone. QS-21 fraction has two major isomeric components; QS-21_{Api} and QS-21_{Xyl} (Soltysik *et al*., 1993), as shown in **Figure S1**.

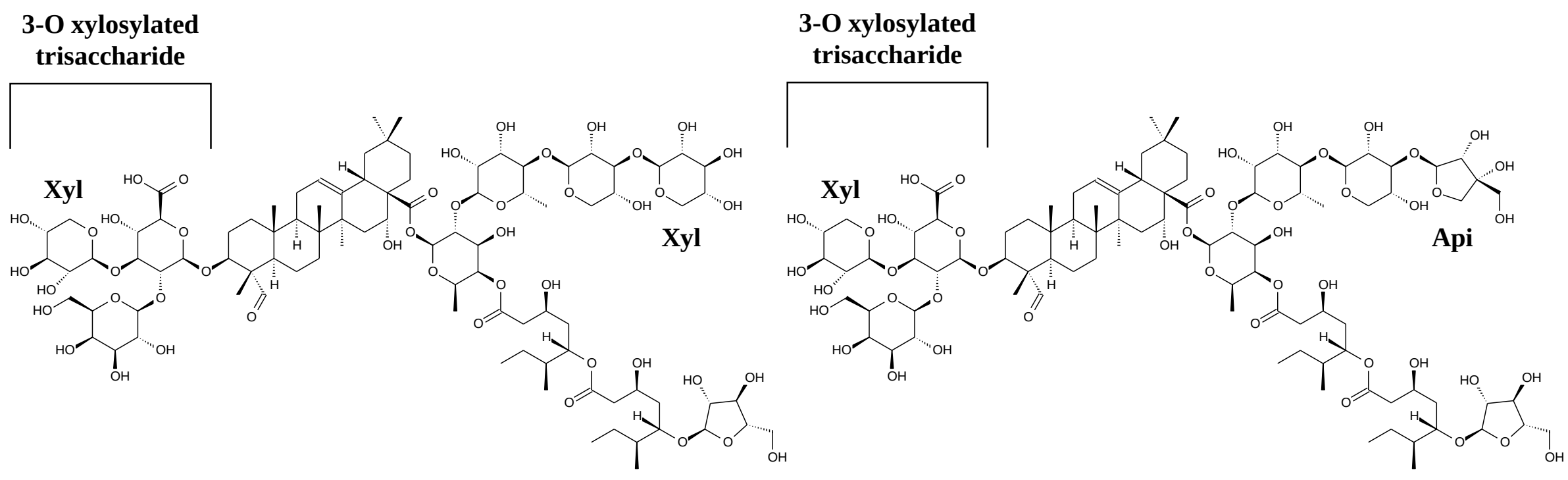

QS-21$_{Xyl}$ QS-21$_{Api}$

Figure S1: Structure of the major isomeric components of QS-21; QS-21$_{Xyl}$ and QS-21$_{Api}$.

Saponin FR is a single peak chromatographic fraction (**Figure S2**) purified by reverse phase HPLC from *Q. saponaria* plants rich in saponins containing the rhamnosylated trisaccharide 3-O-[α-L-rhamnopyranosyl-(1→3)-[β-D-galactopyranosyl-(1→2)]-β-D-glucuronopyranoside] bonded to position C3 of the aglycone.

In our article "The characterization of a rhamnosylated triterpenic saponin from *Quillaja saponaria* Mol. reveals novel adjuvant, antibacterial, antiviral, antifungal and antiproliferative properties" we provide the negative mode ESI mass spectrum of Saponin FR revealing the isotopic cluster of a quasi-molecular ion $[M-H]^-$, 14 Th heavier than the corresponding ions of the saponin QS-21 suggesting the presence of rhamnose instead of xylose in the trisaccharide in Saponin FR 3-O substituent.

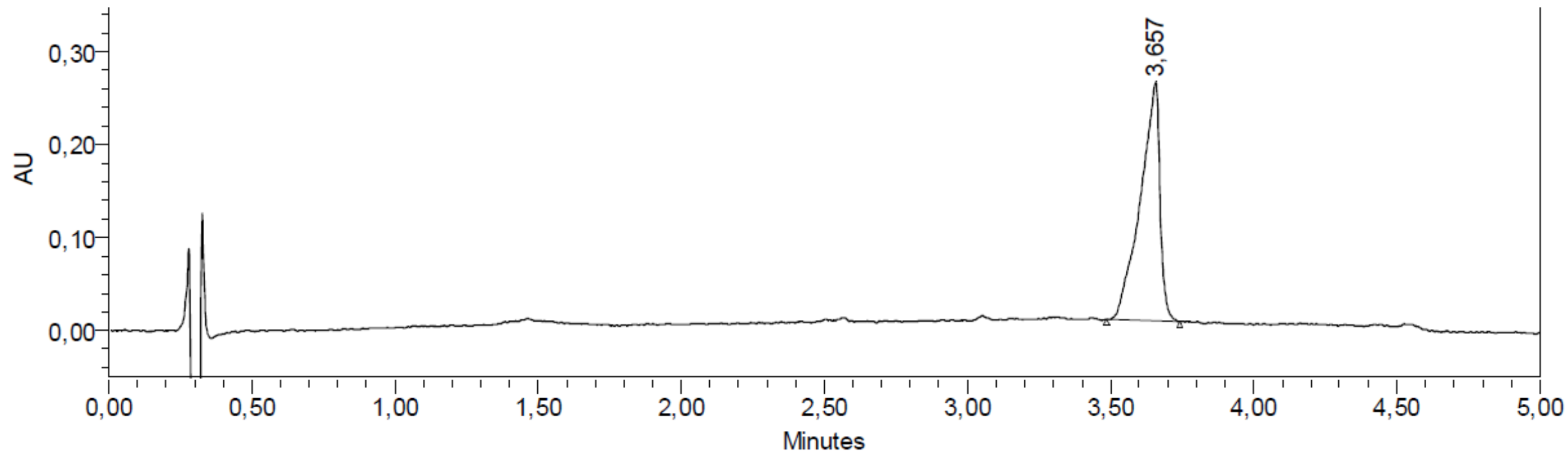

Figure S2: UHPLC chromatogram of Saponin FR. The analytical column used was an Acquity UPLC® BEH C-18 (particle size 1.7 µm, inner diameter 2.1 mm; length 50 mm) (Waters, MA, USA). The column was maintained at 30 °C during each chromatographic run. The injected sample (6 µl of 2 g/l solution in water/acetonitrile 70:30) was eluted at constant flow rate (0.41 ml/min) in gradient elution mode employing the following mobile phases: Solvent A: Formic acid 0.15% v/v in water, and Solvent B: Formic acid 0.15% v/v in acetonitrile. The solvent gradient employed is described as follows: Step 1: 0.0-2.5 min from 34% to 45% of Solvent B; Step 2: 2.5-3.5 min 45% of Solvent B; Step 3: 3.5-5.0 min from 45% to 34% of Solvent B, and Step 4: 5.0-7.0 min 34% of Solvent B. The eluted peaks were detected by monitoring the absorbance at 210 nm.

Based on the recently published biosynthetic pathway of *Q. saponaria* (Martin *et al*., 2024; Reed *et al*., 2023) Saponin FR should contain also two major isomeric components analogs; FR_{Api} and FR_{Xyl}, as shown in **Figure S3**.

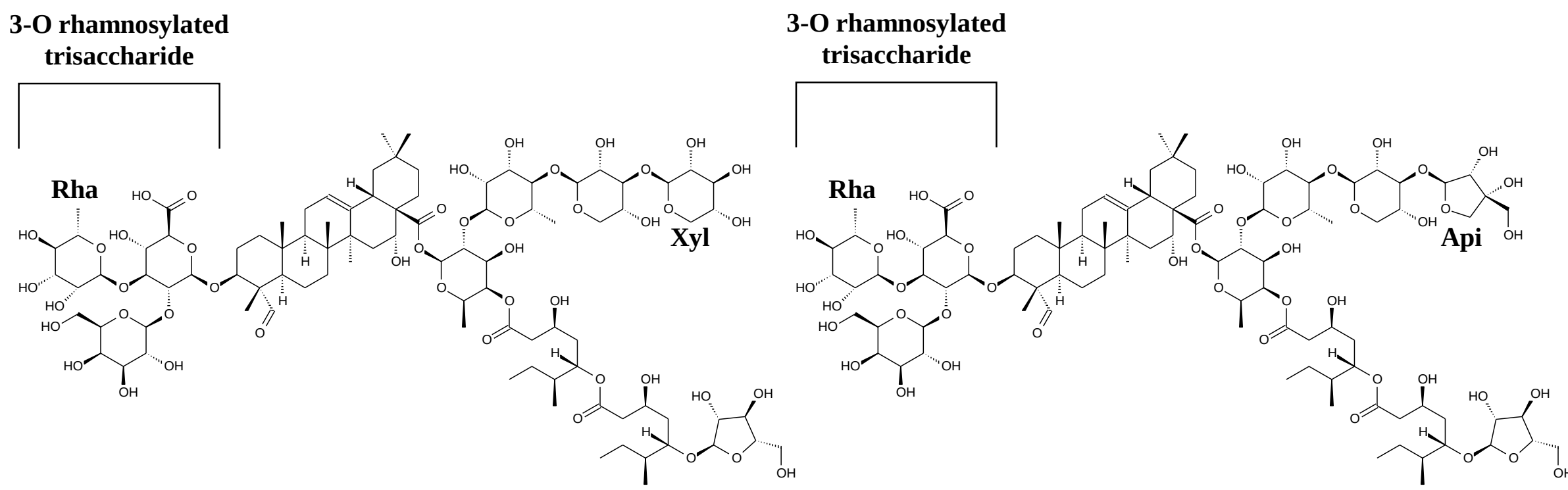

FR_{Xyl} FR_{Api}

Figure S3: Structure of the major isomeric components of Saponin FR; FR_{Xyl} and FR_{Api}.

Nord & Kenne (1999) were able to isolate QS-21_{Xyl}, QS-21_{Api} as well as the proposed FR_{Xyl} and FR_{Api} components from quillaja bark extracts (called S4, S6, S3 and S5 compounds, respectively) which were structurally characterized based on MS and NMR analysis.

In our article "The characterization of a rhamnosylated triterpenic saponin from *Quillaja saponaria* Mol. reveals novel adjuvant, antibacterial, antiviral, antifungal and antiproliferative properties" we provide evidence, based on mass spectrometry data, to support the chemical structure of the major component of Saponin FR. To provide further evidence of the presence of rhamnose in the trisaccharide bonded to position C3 of the aglycone of Saponin FR, we performed additional NMR analysis; a sample of QS-21 was analyzed as well for comparison purposes.

Samples.

Freeze-dried powders of Saponin FR and QS-21 were produced at Desert King Chile. Both saponin fractions were produced by reverse-phase HPLC fractionation of extracts of *Q. saponaria* enriched in each saponin type.

Instrumentation.

Saponin samples were characterized by proton and carbon-13 nuclear magnetic resonance in a Bruker Avance III HD-400 spectrometer. Chemical shifts are expressed in parts per million (δ scale) referenced to the protium residue of the DMSO-d_6 (CD_3SOCD_3) employed as NMR solvent (δ 2.50 for ^{1}H NMR, δ 40.4 for ^{13}C NMR). Both samples (15 mg each) were dissolved in 0.5 ml of deuterated DMSO-d_6 and analyzed by ^{1}H and ^{13}C NMR as well as by 2D NMR (COSY and HSQC). All the analyses were performed at 25 °C.

Analysis of spectroscopic results.

Figures S4 and **S5** show the ^{1}H and ^{13}C NMR of Saponin FR and QS-21.

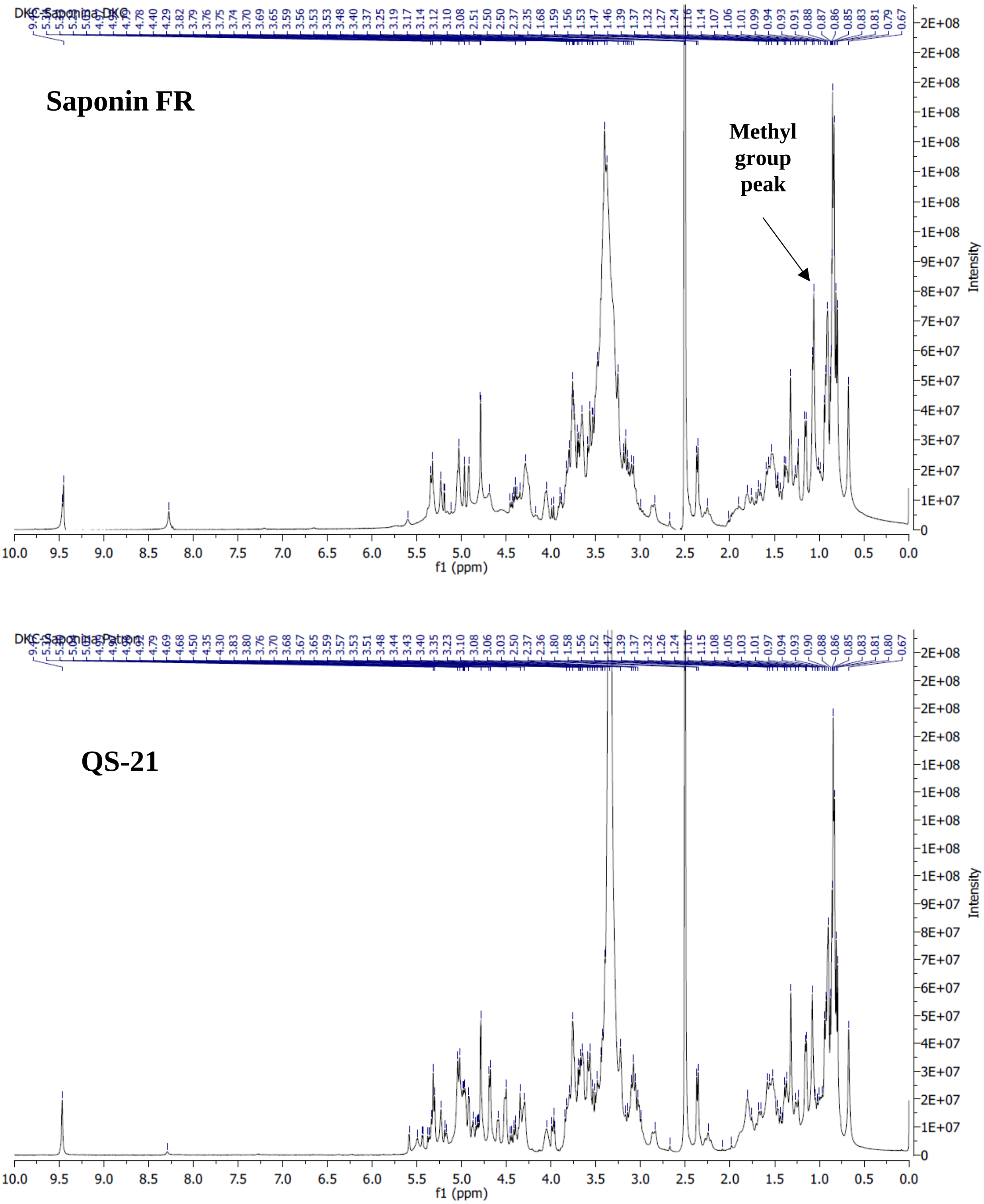

Figure S4: ^{1}H NMR of Saponin FR (top) and QS-21 (bottom) dissolved in DMSO-d_6 ~ 30mg/ml.

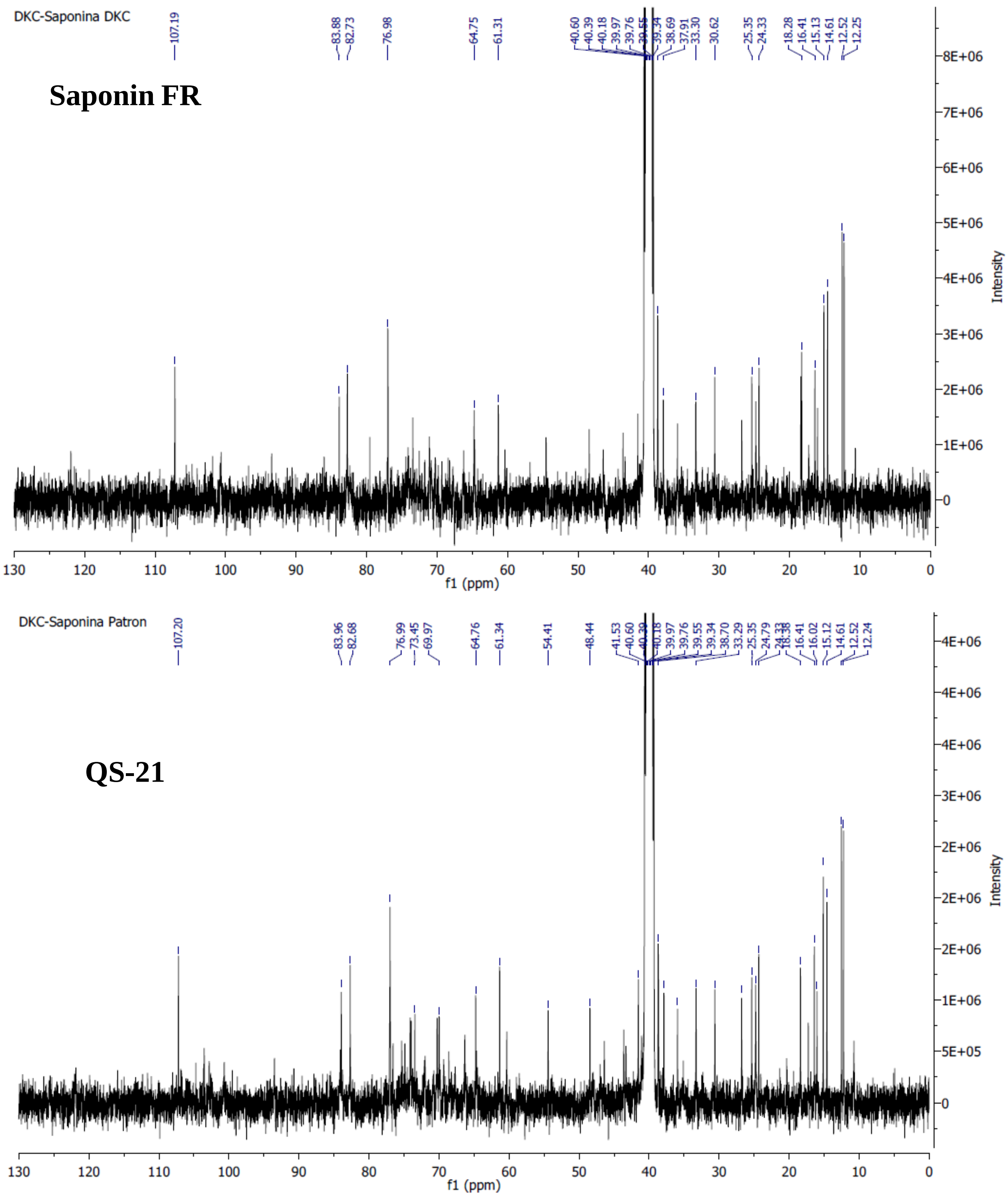

Figure S5: ^{13}C NMR of Saponin FR (top) and QS-21 (bottom) dissolved in DMSO-d_6 ~ 30mg/ml.

Although the ^{1}H NMR reveals a stronger methyl peak (1.06 ppm) in Saponin FR (attributable to the methyl group of rhamnose in the trisaccharide bound to 3-O position of the aglycone), the highly crowded appearance of the spectrum prevents further assignments. To confirm the presence of rhamnose we performed 2D COSY NMR (**Figures S6** and **S8**) and 2D HSQC NMR (**Figures S7** and **S9**) of Saponin FR and its comparison to QS-21 as a reference. As expected, the spectra of Saponin FR and QS-21 are similar with a few different peaks which could be employed to demonstrate the presence of rhamnose instead of xylose in Saponin FR. From ^{1}H NMR and HSQC data is possible to assign the peak at 1.06/18.14 ppm in HSQC spectrum to the methyl group of rhamnose (Rha-C6); when HSQC spectra of Saponin FR and QS-21 are overlapped, that peak is observed only in Saponin FR (**Figure S9**). With that information, cross peaks attributable to the ^{1}H bound to carbons Rha-C2, Rha-C3, Rha-C4, Rha-C5 and Rha-C6 (methyl group) of rhamnose were located as cross-peaks in the COSY spectrum of Saponin FR at (**Figure S8**); 4.26, 3.42, 3.18, 3.89 and 1.06 ppm, respectively. No cross-peak attributable to coupling between protons bound to C2 and C1 (anomeric) was observed; considering that L-rhamnose forms α glycosides in *Q. saponaria* saponins (Nord & Kenne 1999, 2000), a weak coupling is expected between those protons (De Bruyn *et al*., 1976), leading to undetectable cross-peak in COSY spectrum. However, a distinctive peak of anomeric carbon is observed only for Saponin FR in overlapped HSQC spectra of Saponin FR and QS-21 (**Figure S9**); therefore, that signal peak was assigned to Rha-C1. The assignments for ^{1}H and ^{13}C atoms in positions Rha-C1, Rha-C2, Rha-C3, Rha-C4, Rha-C5 and Rha-C6 are 4.97/100.66, 4.26/73.04, 3.42/77.95, 3.18/72.44, 3.89/68.65 and 1.06/18.14 ppm, respectively. As shown in **Table S1**, these values are close to those reported for rhamnose substituent in the saponins S3 and S5 (the proposed identities of FR_{Xyl} and FR_{Api}) dissolved in

methanol-d_4 (Nord & Kenne 1999) – the difference in reported chemical shift is attributable to the different solvent employed in our NMR analysis (DMSO-d_6).

Table S1: Experimental (this study) and reported (Nord & Kenne 1999) chemical shifts of ^{1}H and ^{13}C atoms in rhamnose residue in the trisaccharide bonded to 3-O position of the aglycone of the major component in Saponin FR -Note: Solvents employed in NMR studies: Methanol-d_4 (Nord & Kenne 1999) and DMSO-d_6 (this study).

	Saponin FR (this study)		Saponin S3 (Nord & Kenne, 1999) Proposed FR_{Xyl}		Saponin S5 (Nord & Kenne, 1999) Proposed FR_{Api}	
Position	^{1}H [ppm]	^{13}C [ppm]	^{1}H [ppm]	^{13}C [ppm]	^{1}H [ppm]	^{13}C [ppm]
Rha-C1	4.97	100.7	5.03	103.0	5.04	103.1
Rha-C2	4.26	73.0	4.02	71.9	4.02	72.0
Rha-C3	3.42	78.0	3.65	72.1	3.66	72.1
Rha-C4	3.18	72.4	3.40	73.8	3.41	73.7
Rha-C5	3.89	68.7	3.94	70.4	3.94	70.4
Rha-C6	1.06	18.1	1.24	18.3	1.24	17.7

COSY analysis revealed an additional cross-peaks in Saponin FR spectrum at 3.28/4.27 ppm; the chemical shifts suggest coupling between protons bound to C1(anomeric) and C2 carbons of some sugar (most likely in the glycosyl moiety bonded to 3-O position of aglycone; β-D-galactose, or β-D-glucuronic acid).

The presence of β- D -xylose and β- D -apiose isomers of both Saponin FR and QS-21 can be inferred from the evidence based in the chemical characterization of *Q. saponaria* extracts, as well as on the biosynthesis of quillaja saponins (Martin *et al*., 2024; Nord & Kenne 1999, 2000; Reed *et al*., 2023). However, the relative amounts of these isomers in Saponin FR cannot be discerned based on the available NMR data (in this case we cannot rely on the identification of unique peaks in the spectra of Saponin FR and QS-21 to identify those residues, since both compounds should

show almost identical signals for sugars located in the glycosyl moiety bonded to 28-O position of aglycone). The quantification of the relative amount of those isomers will be a subject of a future publication.

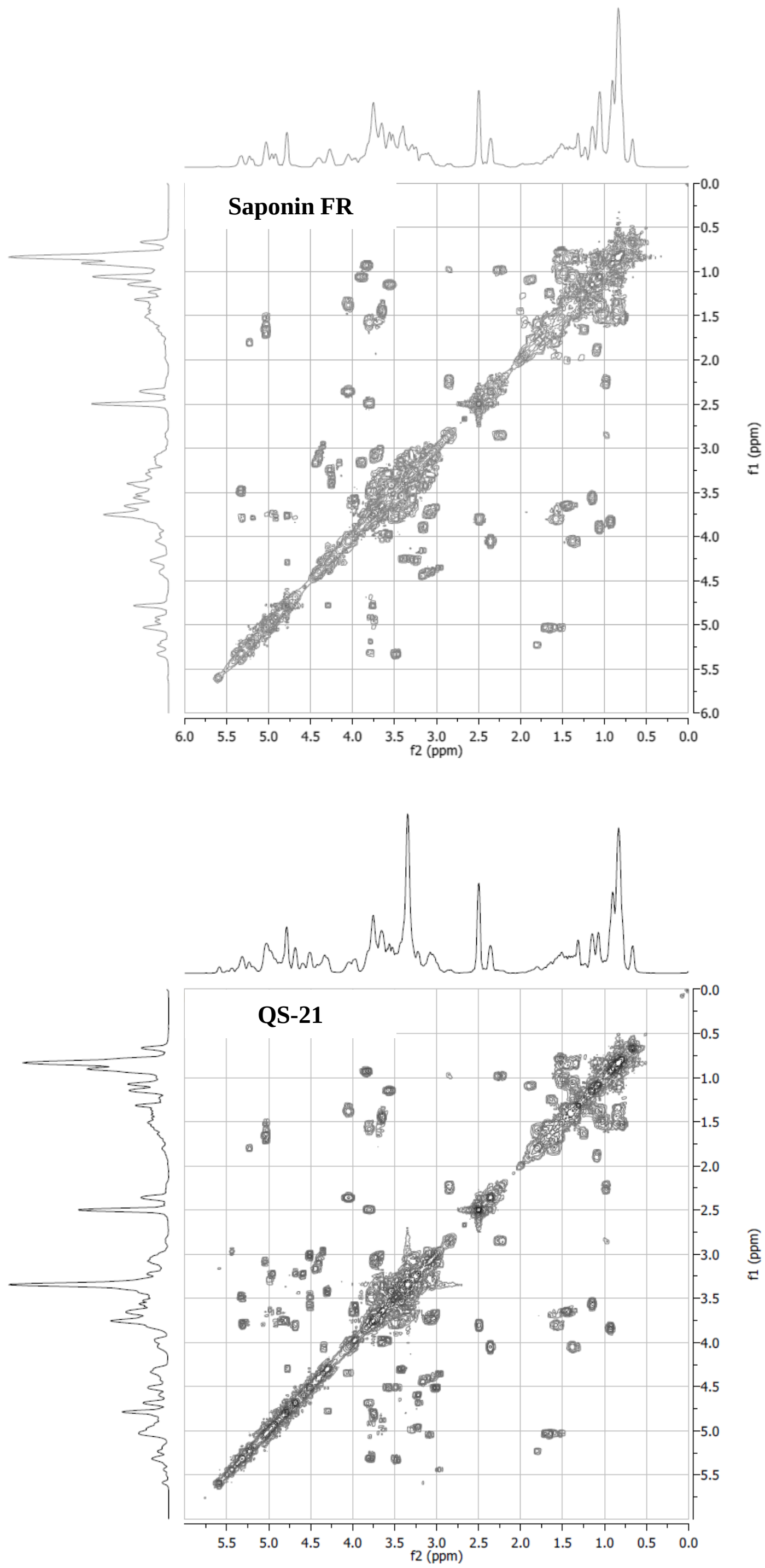

Figure S6: 2D COSY NMR of Saponin FR (top) and QS-21 (bottom) dissolved in DMSO-d_6 ~ 30mg/ml.

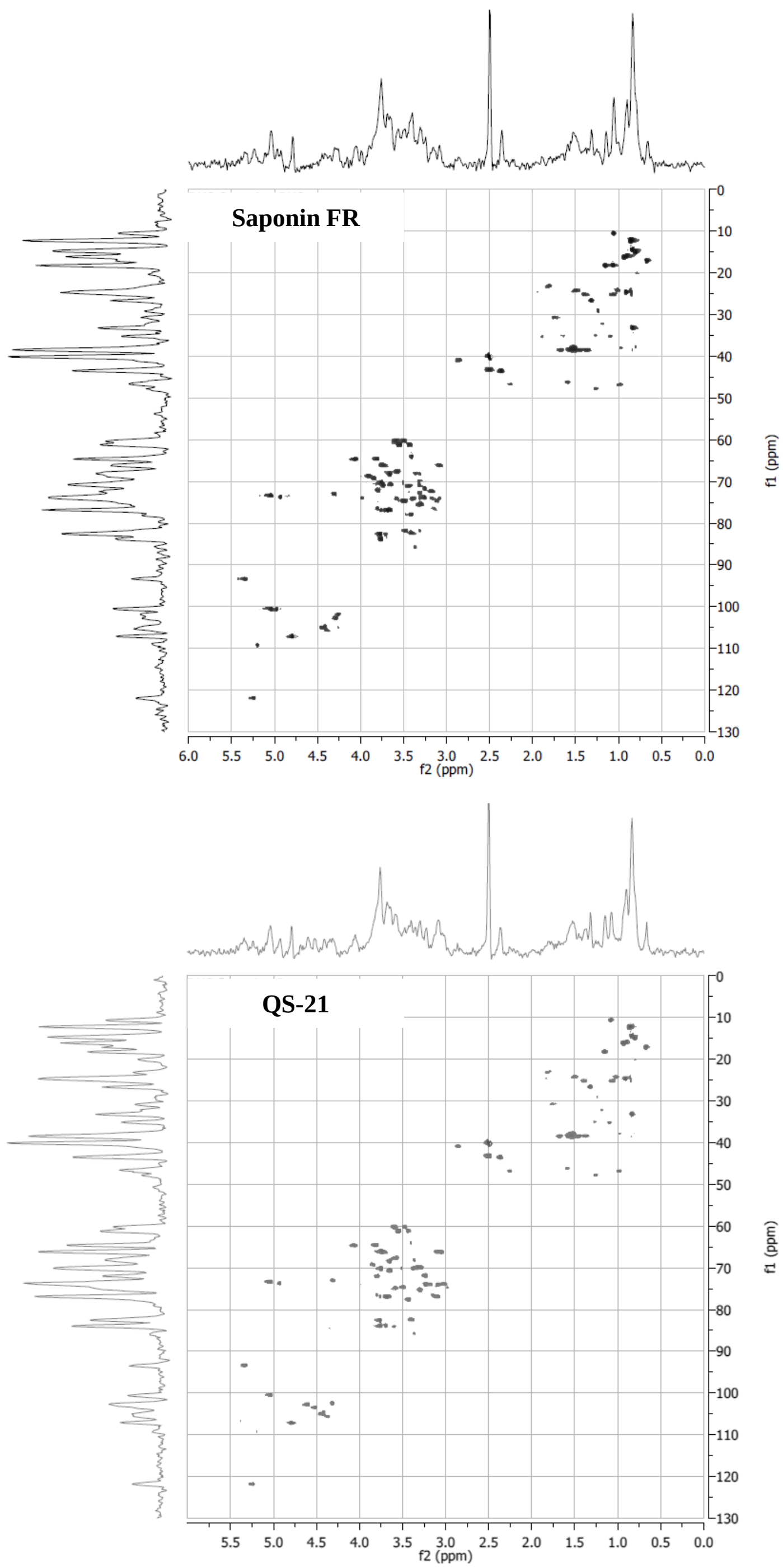

Figure S7: 2D HSQC NMR of Saponin FR (top) and QS-21 (bottom) dissolved in DMSO-d_6 ~ 30mg/ml.

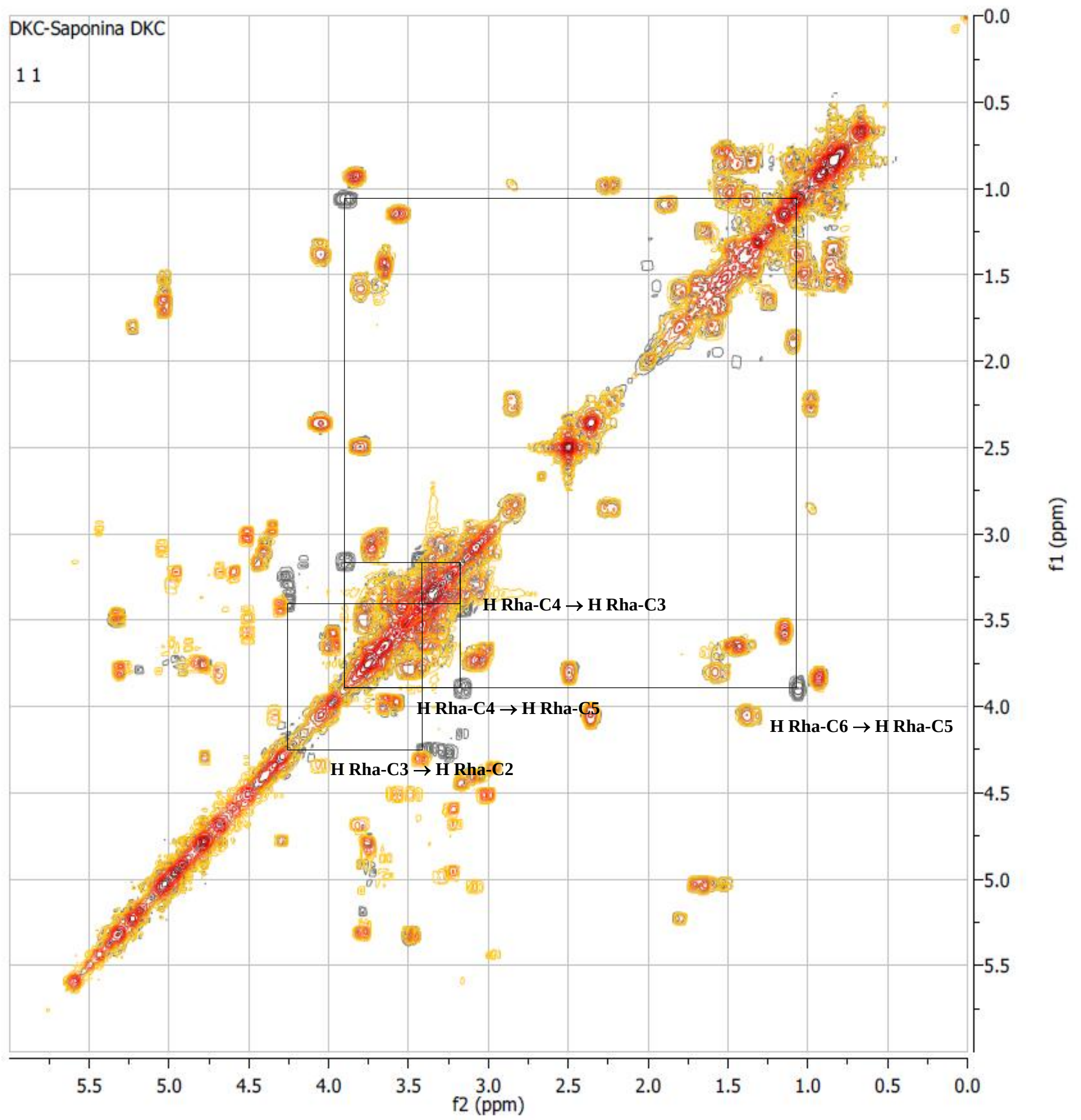

Figure S8: Overlapped 2D COSY NMR of Saponin FR (grey) and QS-21 (colored). Proposed cross peaks attributable to the ^{1}H bound to carbons Rha-C2, Rha-C3, Rha-C4, Rha-C5 and Rha-C6 (methyl group) of rhamnose are shown.

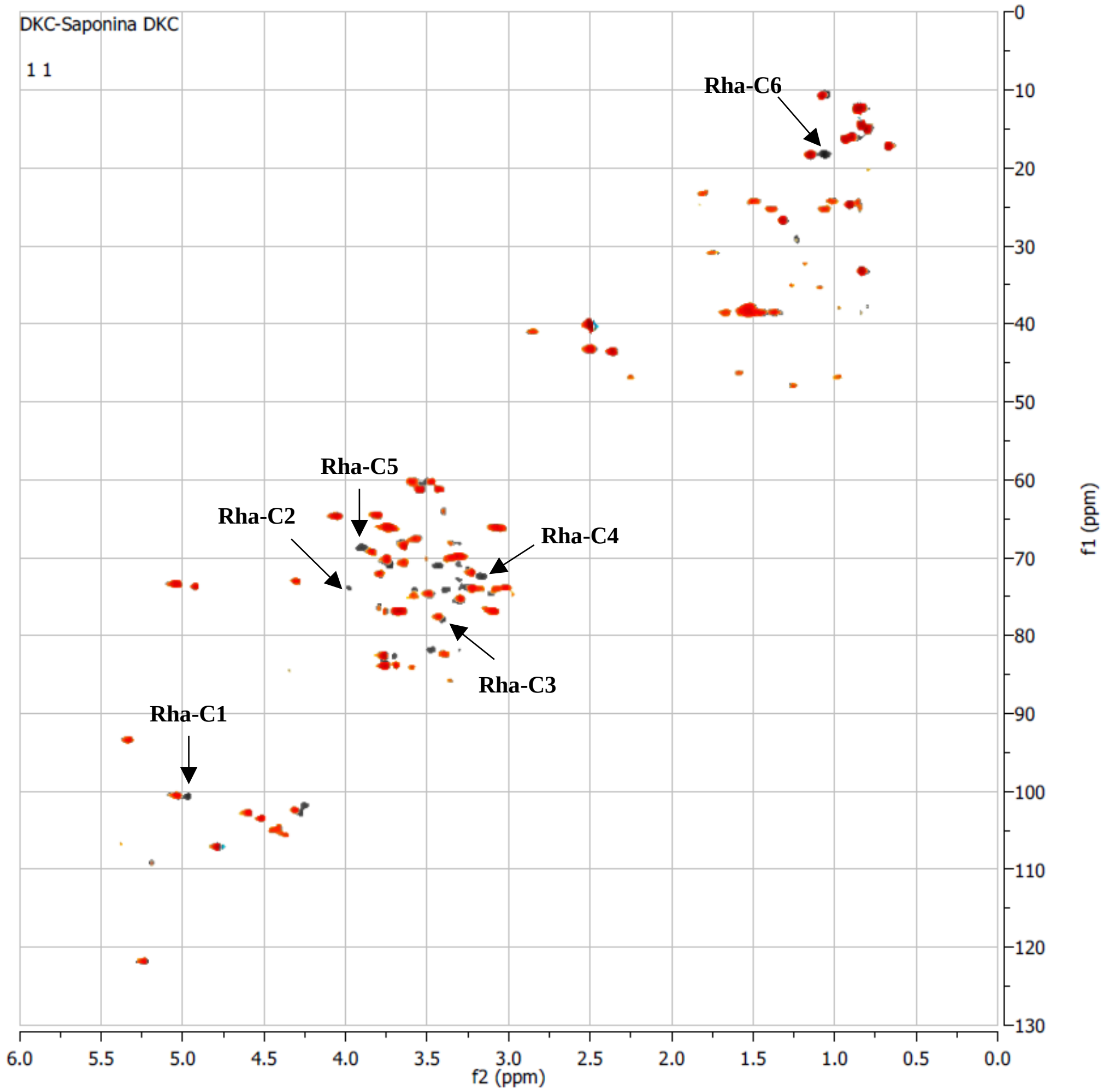

Figure S9: Overlapped 2D HSQC NMR of Saponin FR (black) and QS-21 (red). The assignments for ^{1}H and ^{13}C atoms in positions Rha-C1, Rha-C2, Rha-C3, Rha-C4, Rha-C5 and Rha-C6 are 4.97/100.66, 4.26/73.04, 3.42/77.95, 3.18/72.44, 3.89/68.65 and 1.06/18.14 ppm, respectively.

REFERENCES

1. De Bruyn A, Anteunis M. 1H-N.m.r. study of L-rhamnose, methyl alpha-L-rhamnopyranoside, and 4-O-beta-D-galactopranosyl-L-rhamnose in deuterium oxide. Carbohydr Res (1976) 47:158-63. doi: 10.1016/s0008-6215(00)83559-4.
2. Martin LBB, Kikuchi S, Rejzek M, Owen C, Reed J, Orme A, *et al*. Complete biosynthesis of the potent vaccine adjuvant QS-21. Nat Chem Biol (2024) 20: 493-502. doi: 10.1038/s41589-023-01538-5.

3. Nord LI, Kenne L. Separation and structural analysis of saponins in a bark extract from *Quillaja saponaria* Molina. Carbohydr Res (1999) 320:70-81. doi: 10.1016/s0008-6215(99)00134-2.
4. Nord LI, Kenne L. Novel acetylated triterpenoid saponins in a chromatographic fraction from *Quillaja saponaria* Molina. Carbohydr Res (2000) 329:817-29. doi: 10.1016/s0008-6215(00)00248-2.
5. Reed J, Orme A, El-Demerdash A, Owen C, Martin LBB, Misra RC, *et al*. Elucidation of the pathway for biosynthesis of saponin adjuvants from the soapbark tree. Science (2023) 379:1252-1264. doi: 10.1126/science.adf3727.
6. Soltysik S, Bedore DA, Kensil CR. Adjuvant activity of QS-21 isomers. Ann N Y Acad Sci (1993) 690:392-395. doi: 10.1111/j.1749-6632.1993.tb44041.x.
7. Van Setten DC, van de Werken G, Zomer G, Kersten GF. Glycosyl compositions and structural characteristics of the potential immuno-adjuvant active saponins in the *Quillaja saponaria* Molina extract Quil A. Rapid Commun Mass Spectrom (1995) 9:660-666. doi: 10.1002/rcm.1290090808.
